# Effects of Sodium Butyrate on Digestive Metabolism, Blood Gas Parameters and Blood Biochemical Indices in Tumbler Pigeons Based on Untargeted Metabolomics

**DOI:** 10.3390/ani16131941

**Published:** 2026-06-23

**Authors:** Kunyu Liao, Haiying Li, Xiaobin Li, Xinsheng Guo, Xiaoyu Zhao

**Affiliations:** College of Animal Science, Xinjiang Agricultural University, Urumqi 830052, China; 18915391126@163.com (K.L.); lhy-3@163.com (H.L.); lxb262819@163.com (X.L.); 13017602713@163.com (X.G.)

**Keywords:** sodium butyrate, tumbler pigeon, non-targeted metabolomics, blood gas analysis, blood biochemistry

## Abstract

This study investigated the effects of dietary sodium butyrate supplementation on blood gas profiles, biochemical parameters, and untargeted plasma metabolomics in tumbler pigeons. Eighty tumbler pigeons of similar age, body weight, and training intensity were randomly allocated into four groups: a control group without sodium butyrate supplementation (CON) and three sodium butyrate-supplemented groups receiving 6 mg/d (T1), 12 mg/d (T2), and 18 mg/d (T3), respectively. All birds were maintained under identical husbandry conditions and fed the same basal diet throughout a 40-day experimental period consisting of a 10-day acclimation phase and a 30-day feeding trial. Results showed that dietary sodium butyrate supplementation significantly improved dry matter digestibility, with T2 and T3 exhibiting higher values than CON (*p* < 0.05), while metabolizable energy utilization was significantly increased in T3 compared with CON (*p* < 0.05). Sodium butyrate supplementation also significantly altered several blood gas parameters associated with acid–base balance and gas exchange. In addition, antioxidant enzyme activities were enhanced, with SOD, CAT, GSH-Px, and T-AOC activities significantly increased in supplemented groups compared with CON (*p* < 0.01). Furthermore, sodium butyrate supplementation significantly modulated inflammatory responses, increasing IL-10 concentrations (*p* < 0.01) while decreasing IL-6 and IL-8 levels (*p* < 0.01). Untargeted metabolomic analysis revealed significant alterations in pathways related to lipid metabolism, amino acid metabolism, and inflammatory regulation. In conclusion, dietary sodium butyrate supplementation influenced nutrient utilization, blood physiological parameters, antioxidant capacity, inflammatory status, and plasma metabolic profiles in tumbler pigeons. Among the tested supplementation levels, 18 mg/d sodium butyrate was associated with the most favorable overall physiological responses. These findings provide a basis for future investigations into the physiological and metabolic effects of sodium butyrate supplementation in competitive pigeons.

## Simple Summary

Tumbler pigeons experience considerable physiological stress during training and competition, making nutritional management important for maintaining health and physiological function. Sodium butyrate is a feed additive known to support intestinal health, nutrient absorption, and immune regulation in livestock and poultry, but its effects in tumbler pigeons remain poorly understood. Therefore, this study investigated the effects of dietary supplementation with sodium butyrate at 6, 12, and 18 mg/d on nutrient metabolism, blood physiological parameters, immune responses, and plasma metabolites in tumbler pigeons. The results showed that sodium butyrate supplementation improved nutrient utilization and was associated with changes in blood gas parameters, antioxidant status, inflammatory responses, and plasma metabolic profiles. These findings provide useful information for the nutritional management of tumbler pigeons and offer a basis for future studies on the physiological effects of sodium butyrate supplementation.

## 1. Introduction

The tumbler pigeon, also known as the somersault pigeon or the somersaulting pigeon, belongs to the family Columbidae within the order Columbiformes. On the Origin of Species indicates that the tumbler pigeon is a rare breed with distinctive behavioral traits developed through long-term selective breeding from the rock pigeon and its geographic subspecies [1]. As a specialized athletic bird, the tumbler pigeon has attracted growing attention for its unique aerial performance and high exercise demands. The defining characteristic of the acrobatic pigeon is its ability, through systematic training, to perform precise aerial maneuvers—such as somersaults, spins, and vertical dives—in unique formations during flight. As a distinctive racing pigeon breed that combines ornamental and competitive value, it possesses physiological characteristics such as a slender build, delicate bone structure, explosive wing muscle power, and exceptional cardiac output [2], which enable high-frequency, short-duration, high-intensity acrobatic maneuvers suited to the athletic demands of acrobatic tumbler events. However, because this unique mode of exercise relies primarily on instantaneous explosive power, acrobatic pigeons are subjected to sustained high exercise loads during intensive training. This can lead to accelerated energy metabolism, disturbances in acid-base balance, and disruptions in internal homeostasis [3,4], thereby affecting flight performance and post-race recovery capacity [5]. Long-term exercise stress not only causes muscle spasms, joint swelling, decreased endurance, and immunosuppression [6] but may also induce mental agitation, reduced feed intake, and abnormal behavioral manifestations, thereby significantly diminishing training effectiveness and competitive potential [7]. Concurrently, exercise stress activates systemic inflammatory responses and promotes the production of reactive oxygen species [8], leading to damage to muscle fibers and mucosal cells [9]. Under stress, blood flow is prioritized to motor organs such as wing muscles [10], resulting in a relatively insufficient blood supply to the respiratory and intestinal mucosa, thereby compromising the integrity of the mucosal barrier [11]. This predisposes the birds to local inflammatory responses and intestinal dysfunction, thereby impairing nutrient absorption efficiency and exacerbating metabolic disorders and disease risk [12]. In tumbler pigeons, the major physiological stressors associated with intensive training and competition include exercise-induced metabolic stress, oxidative stress resulting from increased reactive oxygen species production, inflammatory responses, and disturbances in acid–base and electrolyte homeostasis. These stressors may collectively impair nutrient utilization, physiological recovery, and metabolic adaptation during prolonged training periods. Given that the physiological status and exercise-related metabolic adaptation of tumbler pigeons are closely associated with nutritional regulation, exploring safe and effective nutritional intervention strategies to alleviate exercise-induced stress and maintain physiological homeostasis has become an important research focus in the scientific feeding and exercise management of tumbler pigeons.

Sodium butyrate, a commonly used supplemental form of short-chain fatty acids, has been widely used in research on livestock and poultry production for its potential to improve function, regulate energy, and modulate responses [13]. Supplementing diets with sodium butyrate or its coated formulations can improve growth performance and health status in livestock and poultry; its mechanisms of action are largely associated with optimizing intestinal structure, enhancing nutrient absorption, and improving metabolic status [14]. Previous studies have also demonstrated that sodium butyrate may enhance antioxidant capacity, regulate immune responses, and influence host metabolic pathways [15,16]. Blood gas parameters reflect the body’s acid–base balance, electrolyte homeostasis, and oxygenation status, all of which are particularly sensitive to physiological disturbances induced by intensive exercise [15]. Exercise-induced oxidative stress and inflammatory responses are closely associated with alterations in tissue oxygen utilization and acid–base regulation. Given the established roles of sodium butyrate in modulating energy metabolism, antioxidant capacity, and inflammatory status, it may also influence physiological processes related to blood gas regulation. Furthermore, untargeted blood metabolomics helps elucidate the effects of nutritional regulation on overall metabolic characteristics [16]. Because blood gas analysis reflects physiological responses to exercise stress, whereas metabolomic profiling provides information on underlying metabolic alterations, integrating these approaches may provide a more comprehensive understanding of the biological responses associated with sodium butyrate supplementation [17]. However, existing studies have primarily focused on production poultry such as broilers and laying hens, with research indicators centered on intestinal morphology, immune function, and antioxidant parameters. Studies on blood gas and blood physiological status in birds under high exercise loads remain relatively limited. In particular, for athletic birds such as racing pigeons, systematic evaluations of the effects of sodium butyrate on blood gas status, blood biochemical characteristics, and systemic metabolic levels remain lacking, which, to some extent, limits its scientific application in the sports nutrition of racing pigeons. Accordingly, this study used tumbler pigeons as the research subjects to systematically investigate the effects of dietary sodium butyrate supplementation on blood gas parameters, plasma biochemical characteristics, and plasma metabolomic profiles. By integrating physiological indicators with metabolomic analysis, this study aimed to provide a more comprehensive evaluation of the potential regulatory effects of sodium butyrate on exercise-related physiological and metabolic responses in athletic pigeons. The aim of this study was to provide a scientific basis for the nutritional regulation of physiological and metabolic responses associated with training and competition in tumbler pigeons, and to offer theoretical guidance for the development and application of functional nutritional supplements.

## 2. Materials and Methods

### 2.1. Animal Ethics Statement

This study was reviewed and granted by the Institutional Animal Care and Use Ethics Committee of Xinjiang Agricultural University (Urumqi, China; protocol permit number: 2023008).

### 2.2. Animals and Experimental Design

This study selected 80 tumbler pigeons aged 2–3 years and weighing 280 ± 30 g, with similar training regimens and intensities, provided by the Lifen Rare Bird Cooperative in Yanqi County, Xinjiang. The pigeons were randomly assigned to four treatment groups (20 birds per treatment). Each treatment consisted of 10 replicates, with 2 pigeons housed together in each replicate. Randomization was performed to ensure that birds of similar age, body weight, and training history were evenly represented among treatments. The replicate structure was maintained throughout the experimental period. For subsequent laboratory analyses, one pigeon was randomly selected from each replicate as the representative bird for sample collection. The groups were designated as the control group, T1, T2, and T3. Under identical exercise training and feeding management conditions, using an isocaloric, isonitrogenous diet as the baseline, the CON received no sodium butyrate supplementation, while T1, T2, and T3 received 6 mg/d, 12 mg/d, and 18 mg/d of sodium butyrate per animal, respectively. A 40-day feeding and exercise training trial was conducted, consisting of a 10-day adaptation period followed by a 30-day experimental period. During the adaptation period, all pigeons were maintained under identical feeding and training conditions and received only the basal diet to allow acclimation to the housing environment and management procedures. During the subsequent 30-day experimental period, sodium butyrate supplementation was administered according to the assigned treatment groups, while the same feeding and exercise training program was maintained for all birds. All physiological, biochemical, blood gas, and metabolomic samples were collected at the end of the experimental period. The sodium butyrate supplementation levels were selected according to previous studies evaluating dietary sodium butyrate in poultry and pigeons, as well as the manufacturer’s recommended inclusion levels, to cover low, moderate, and high supplementation ranges while ensuring dietary safety and practical applicability. During the entire experimental period, pigeons were fed exclusively the basal grain-based diet shown in Table 1. No vitamin–mineral premixes, mineral grit, or other nutritional supplements were provided, in order to minimize potential confounding effects arising from additional nutrient supplementation and to ensure consistency among treatment groups.

During the trial, the study was conducted in accordance with the feeding and management practices for tumbler pigeons at the Yanqi County Lifeng Rare Bird Cooperative. Pigeons in each group underwent two training flights daily: the first at 9:00 AM and the second at 5:00 PM, each lasting 30 min. After each training session, the pigeons were fed 9 g of the basal diet. Following the second daily feeding, sodium butyrate was administered individually in capsule form to ensure accurate dosage delivery. Each pigeon received the assigned amount of sodium butyrate (6, 12, or 18 mg/d) once daily throughout the experimental period. The experimental pigeons were maintained in a consistent rearing environment; cages were cleaned and disinfected regularly after each feeding, and clean drinking water was provided. The temperature in the pigeon loft was maintained at approximately 11 °C. Animal welfare was monitored throughout the experimental period. All pigeons were inspected daily by trained personnel for general health status, feed and water intake, behavioral activity, feather condition, and signs of injury or disease. Additional observations were conducted before and after training sessions and competition events to identify potential indicators of excessive fatigue, distress, or physical injury. Any bird exhibiting severe health abnormalities would have been withdrawn from the experiment and provided appropriate veterinary care. No mortality, severe injury, or other adverse welfare events were observed during the study.

Nutritional components were determined according to Chinese national standards. Crude protein (CP) was determined using the Kjeldahl method (GB/T 6432-1994) [18]. Ether extract (EE) was analyzed using the Soxhlet extraction method (GB/T 6433-2006) [19]. The methods for determining neutral detergent fiber (NDF) and acid detergent fiber (ADF) were described by Van Soest et al. [20]. Calcium and phosphorus contents were determined using the methods specified in GB/T 6437-2002 [21]. The crude protein content was 18.34%, crude fat content was 15.31%, NDF content was 24.90%, ADF content was 2.64%, calcium content was 0.38%, and phosphorus content was 0.13%. Table 1 shows the composition and nutritional components of the basal diet.

### 2.3. Sample Collection and Processing

On day 30 of the experimental period, a simulated race was conducted in conjunction with the Yanqi Tumbler Pigeon Competition organized by the Yanqi County Tumbler Pigeon Association.

For plasma biochemical, antioxidant, and inflammatory analyses, one pigeon was randomly selected from each of the 10 replicates in every treatment group, resulting in 10 biological samples per treatment group.

For blood gas analysis, ten replicates were randomly allocated to either pre-exercise (BF) or post-exercise (AF) sampling. Different individuals were used for BF and AF measurements to avoid potential effects of repeated blood collection. Therefore, five biological samples were obtained for each sampling condition within each treatment group.

Prior to blood collection, pigeons were gently restrained by trained personnel, and the sampling site was disinfected with 75% ethanol. Fresh venous blood was collected from the brachial vein using sterile vacuum blood collection tubes (Jiangsu Kangjian Medical Instrument Co., Ltd., Taizhou, Jiangsu, China). Approximately 1 mL of fresh whole blood was obtained using a non-heparinized syringe and immediately analyzed using a veterinary blood gas analyzer (VG2, Seamaty Technology Co., Ltd., Chengdu, China) according to the manufacturer’s instructions. Blood samples were analyzed immediately after collection, and the average sampling and analytical time for each pigeon was approximately 2–3 min.

For plasma biochemical, antioxidant, inflammatory, and metabolomic analyses, approximately 3 mL of venous blood was collected from the brachial vein into sodium-heparin anticoagulant tubes (Jiangsu Kangjian Medical Instrument Co., Ltd., Taizhou, Jiangsu, China). Samples were centrifuged at 4000 rpm for 5 min, and approximately 1.5 mL of plasma was transferred into sterile cryogenic tubes plasma samples were rapidly frozen in liquid nitrogen and stored at −80 °C until further analysis. The total blood volume collected from each pigeon remained within the recommended limits for avian blood sampling, and no adverse effects associated with blood collection were observed during the experiment.

For untargeted metabolomic analysis, five pigeons were randomly selected from five independent replicates within each treatment group. Based on the overall physiological responses observed in the present study and preliminary evidence from a parallel gut microbiota investigation, the T2 group was considered a representative sodium butyrate supplementation level and was therefore selected for metabolomic profiling to explore potential metabolic alterations associated with sodium butyrate supplementation.

### 2.4. Sampling and Determination of Nutrient Digestion and Metabolism Rates

During days 26–30 of the experimental period, apparent nutrient digestibility was determined using the total feces collection method. Each treatment consisted of 10 replicates, with two pigeons assigned to each replicate. For the digestibility trial, both pigeons within each replicate were individually housed in metabolic cages to allow accurate collection of feed intake and fecal output. Prior to sample collection, the pigeons were fasted for 24 h to empty the gastrointestinal tract. Subsequently, each pigeon was offered 18 g of the experimental diet daily for 3 consecutive days. Feed intake was recorded daily throughout the collection period. All feces excreted during the 3-day collection period were collected quantitatively from each pigeon. The feed and air-dried feces samples were oven-dried at 65 °C to constant weight, ground, and sieved through a fine mesh for chemical analysis. Fecal samples were air-dried prior to oven drying to prevent crust formation, minimize the loss of volatile compounds, and inhibit microbial activity, thereby ensuring the accuracy of subsequent chemical analyses.

Determine the DM, organic matter (OM), ME, CP, and EE content in the fecal samples, and determine the DM, OM, Gross energy (GE), CP, EE, and crude fiber (CF) content in the feed samples. Analytical methods followed Chinese national standards: DM: GB/T 6435-2014 [22]; OM: GB/T 6438-2007 [23]; GE: GB/T 14489.1-2008 [24]; CP: GB/T 6432-1994 [18]; EE: GB/T 6433-2006 [19]. The formula for calculating metabolizable energy is ME (kcal/kg DM) = [GE intake − (GE in feces + GE in urine)]/feed DM intake.

### 2.5. Determination of Indicators and Methods

Blood gas and electrolyte parameters were determined using a veterinary blood gas analyzer (VG2, Seamaty Technology Co., Ltd., Chengdu, China) equipped with disposable dry electrochemical cartridges according to the manufacturer’s instructions. Immediately after blood collection, whole blood samples were loaded into the test cartridges and analyzed without delay to minimize alterations caused by storage. The measured blood gas parameters included pH, partial pressure of carbon dioxide (PvCO_2_), partial pressure of oxygen (PvO_2_), sodium (Na^+^), potassium (K^+^), chloride (Cl^−^), ionized calcium (Ca^2+^), hematocrit (Hct), total carbon dioxide (TCO_2_), bicarbonate (HCO^3−^), extracellular fluid base excess [BE(ecf)], blood base excess [BE(b)], hemoglobin (Hb), and oxygen saturation (SvO_2_). The average time required for blood collection and analysis was approximately 2–3 min per pigeon. All test results were automatically uploaded to a computer for subsequent statistical analysis.

Plasma biochemical parameters were determined using an automatic biochemical analyzer (BS-420, Mindray Bio-Medical Electronics Co., Ltd., Shenzhen, China) with commercial reagent kits supplied by Biosino Bio-technology and Science Incorporation (Beijing, China). Analyses were performed by Beijing Huaying Biotechnology Research Institute (Beijing, China). The biochemical parameters included total protein (TP), albumin (ALB), globulin (GLB), albumin-to-globulin ratio (A/G), aspartate aminotransferase (AST), alanine aminotransferase (ALT), alkaline phosphatase (ALP), total cholesterol (TC), triglycerides (TG), glucose (GLU), lactate (LAC), γ-glutamyltransferase (γ-GT), lactate dehydrogenase (LDH), creatine kinase (CK), and uric acid (UA).Plasma immunological parameters included immunoglobulin M (IgM), immunoglobulin G (IgG), and immunoglobulin A (IgA). Plasma antioxidant parameters included superoxide dismutase (SOD), catalase (CAT), glutathione peroxidase (GSH-Px), malondialdehyde (MDA), and total antioxidant capacity (T-AOC). Plasma inflammatory parameters included tumor necrosis factor-α (TNF-α), interleukin-1β (IL-1β), interleukin-6 (IL-6), interleukin-10 (IL-10), interferon-γ (IFN-γ), interleukin-8 (IL-8), interleukin-4 (IL-4), and interleukin-18 (IL-18). Immunological, antioxidant, and inflammatory parameters were measured using commercial assay kits purchased from Nanjing Jiancheng Bioengineering Institute (Nanjing, China) according to the manufacturer’s instructions. Absorbance values were determined using a DR-200BS microplate reader (Hwato Delang Medical Equipment Co., Ltd., Beijing, China).

### 2.6. Metabolite Extraction, UHPLC-MS/MS, and Metabolomics Analysis

This study employed liquid chromatography–mass spectrometry (LC–MS/MS)-based untargeted metabolomics to characterize plasma metabolic profiles [25,26]. Quality control (QC) samples were prepared by pooling equal aliquots from all plasma samples and were analyzed before, during, and after sample acquisition to monitor instrument performance and analytical reproducibility. The initial QC injections were used to equilibrate the chromatographic and mass spectrometric systems, whereas QC samples interspersed throughout the analytical sequence were used to evaluate system stability and data quality.

Raw mass spectrometry data were converted into mzXML format using ProteoWizard software, version 3.0 [27], and processed using XCMS software, version 3.2 [28], for peak detection, retention time correction, peak alignment, and peak quantification. Peaks with a missing value rate greater than 50% were removed prior to downstream analysis. The resulting data were normalized based on total peak area. Metabolite annotation was performed by matching accurate mass information, retention time, and MS/MS fragmentation spectra against the Novogene in-house metabolite database. Metabolite identification confidence was evaluated according to the Metabolomics Standards Initiative (MSI) guidelines. Most annotated metabolites were assigned to MSI Level 2 based on accurate mass and MS/MS spectral matching, whereas metabolites lacking MS/MS confirmation were classified as MSI Level 3. To ensure data reliability, only metabolites with a coefficient of variation (CV) < 30% [29] in QC samples were retained for subsequent analyses.

Multivariate statistical analyses, including principal component analysis (PCA) and partial least squares discriminant analysis (PLS-DA), were conducted to evaluate metabolic differences among groups. Hierarchical cluster analysis (HCA), metabolite correlation analysis, and KEGG pathway enrichment analysis were further performed to explore biological functions associated with differential metabolites. Model robustness and potential overfitting were assessed using permutation testing. Differential metabolites were identified using the criteria of variable importance in projection (VIP) > 1.0, fold change (FC) > 1.2 or <0.833, and *p* < 0.05. KEGG pathway enrichment analysis was performed using a hypergeometric test, and the resulting *p* values were adjusted for multiple comparisons using the Benjamini–Hochberg false discovery rate (FDR) correction method.

The reliability and stability of the LC-MS/MS system were evaluated using pooled QC samples. Pearson correlation coefficients among QC samples exceeded 0.99 in both positive and negative ion modes, and QC samples were tightly clustered in PCA score plots, indicating excellent analytical reproducibility and instrument stability throughout the analytical sequence.

### 2.7. Data Processing and Statistical Analysis

Prior to statistical analysis, data were tested for normality using the Shapiro–Wilk test and for homogeneity of variances using Levene’s test. After confirming that the assumptions of ANOVA were satisfied, data were analyzed using one-way analysis of variance (ANOVA) in IBM SPSS Statistics software, version 27.0 (IBM Corp., Armonk, NY, USA) to assess differences between the control group and each experimental group. Subsequently, Duncan’s multiple range test was used to perform multiple comparisons among the groups. Results are expressed as mean ± standard deviation (Mean ± SD). A *p* < 0.05 was considered statistically significant, and a *p* < 0.01 was considered highly significant.

## 3. Results

### 3.1. Effects of Supplemental Feeding of Sodium Butyrate on Apparent Metabolic Rate of Nutrients in Tumbler Pigeons

As shown in Table 2, regarding the apparent metabolic rate of DM, T2 and T3 was significantly higher than CON (*p* < 0.05). For ME apparent metabolic rate, T3 was extremely significantly higher than the control group (*p* < 0.01), T2 was significantly higher than that of the control group (*p* < 0.05). There were no significant differences in the apparent metabolic rates of OM, CP, and EE among the groups (*p* > 0.05).

### 3.2. The Effects of Sodium Butyrate Supplementation on Blood Gas Parameters in Tumbler Pigeons Before and After Competition

**Figure 1:**
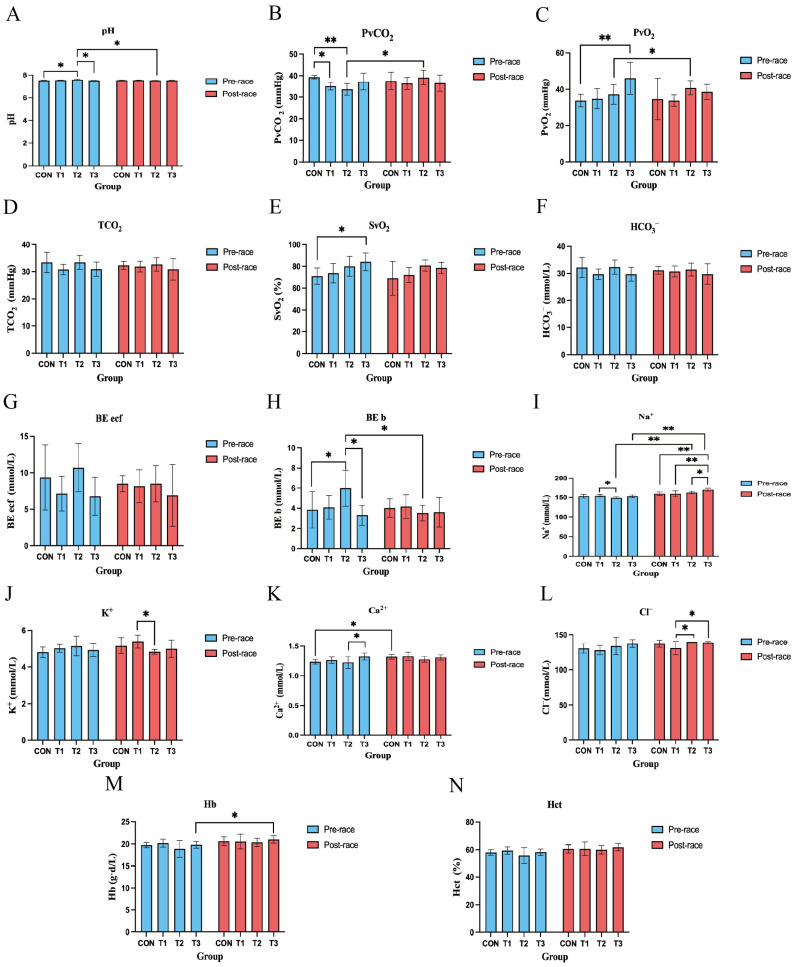
Effects of sodium butyrate on blood gas profiles in tumbler pigeons pre- and post-race. (**A**) pH; (**B**) Partial pressure of carbon dioxide (PvCO_2_); (**C**) Partial pressure of oxygen (PvO_2_); (**D**) Total carbon dioxide (TCO_2_); (**E**) Venous oxygen saturation (SvO_2_); (**F**) Bicarbonate ion (HCO_3_^−^); (**G**) Base excess of extracellular fluid (BEecf); (**H**) Base excess (BEb); (**I**) Sodium ion (Na^+^); (**J**) Potassium ion (K^+^); (**K**) Calcium ion (Ca^2+^); (**L**) Chloride ion (Cl^−^); (**M**) Hemoglobin (Hb); (**N**) Hematocrit (Hct). Blue bars represent pre-race measurements, and red bars represent post-race measurements. Data are presented as mean ± standard error (SE). * *p* < 0.05 indicates a statistically significant difference; ** *p* < 0.01 indicates an extremely significant difference.

Detailed data on changes in blood gas parameters in tumbler pigeons before and after competition following supplementation with sodium butyrate are shown in Figure 1 (Table S1). Overall, dietary sodium butyrate supplementation resulted in relatively limited alterations in blood gas variables. No significant effects were observed on major acid–base buffering parameters (HCO_3_^−^ and BEecf), gas exchange-related TCO_2_, or hematocrit (Hct) (*p* > 0.05), although several individual blood gas and electrolyte variables showed statistically significant differences among treatments. The pH value in the T2 group before the race was significantly higher than that in the control group and the T3 group and was also significantly higher than that in the post-race T2 group (*p* < 0.05). However, no significant differences in pH were observed among treatment groups after the race (*p* > 0.05). Pre-exercise SvO_2_ levels in the T3 group were significantly higher than those in the CON group (*p* < 0.05), whereas no significant post-exercise differences were detected among treatments. Pre-race levels in the T2 were extremely significantly lower than those in the CON (*p* < 0.01) and significantly lower than those in the post-race T2 (*p* < 0.05). Post-race PvCO_2_ levels did not differ significantly among (*p* > 0.05). Pre-exercise PvO_2_ levels in the T3 were extremely significantly higher than those in CON (*p* < 0.01). There were no significant differences in PvO_2_ levels among groups post-exercise (*p* > 0.05), and pre-exercise levels in the T2 were significantly lower than those in the post-exercise T2 (*p* < 0.05). Pre-exercise SvO_2_ levels in the T3 were significantly higher than those in the CON (*p* < 0.05). BEb levels in the T2 were significantly higher than those in the CON and T3 (*p* < 0.05) and were also significantly higher than those in the post-race T2 (*p* < 0.05); there were no significant differences among the other (*p* > 0.05). Na^+^ levels in the pre-race T2 were significantly lower than those in the pre-race T1 (*p* < 0.05) and extremely significantly lower than those in the post-race T2 (*p* < 0.01). The post-race T3 had extremely significantly higher levels than the post-race CON, T1, and pre-race T3 (*p* < 0.01), and significantly higher levels than the post-race T2. K^+^ levels in the post-race T1 were significantly higher than those in the post-race T2 (*p* < 0.05). Ca^2+^ levels in the pre-race T3 were significantly higher than those in the T2 (*p* < 0.05), and levels in the post-race CON were significantly higher than those in the pre-race CON (*p* < 0.05). Post-race Cl^−^ levels in the T1 were significantly lower than those in the T2 and T3 (*p* < 0.05). Post-race Hb levels in the T3 were significantly higher than those in the pre-race T3 (*p* < 0.05).

Although several electrolyte variables (Na+, K+, Ca2+, and Cl^−^) differed significantly among treatment groups, the magnitude of these changes was generally modest and no consistent treatment-related pattern was observed across sampling periods.

### 3.3. Effects of Sodium Butyrate Supplementation on Plasma Biochemical, Antioxidant, Immune, Inflammatory, and Enzyme Parameters in Tumbler Pigeons

The detailed data on the effects of sodium butyrate supplementation on plasma protein metabolism in tumbler pigeons are shown in Table 3 and Table S2. There were no significant differences in TP levels, GLB levels, the albumin-to-globulin (A/G) ratio, or UA levels among all groups (*p* > 0.05). ALB levels in T1 were significantly lower than those in CON *(p* < 0.05).

Regarding plasma antioxidant indicators, there were no significant differences in MDA levels among the groups (*p* > 0.05). For SOD, there was no significant difference between T1 and CON (*p* > 0.05), while T2 and T3 were both significantly higher than CON (*p* < 0.01). For CAT, T1, T2, and T3 were all significantly higher than CON (*p* < 0.01). In the GSH-PX assay, T1, T2, and T3 were all significantly higher than CON (*p* < 0.01). Regarding T-AOC, T1, T2, and T3 were all extremely significantly higher than CON (*p* < 0.01).

Regarding immune markers, there were no significant differences in immunoglobulin M (IgM), immunoglobulin G (IgG) and immunoglobulin A (IgA) levels among the groups (*p* > 0.05).

Regarding plasma anti-inflammatory factors, IL-4 levels were significantly higher in T1 than in CON (*p* < 0.05), and both T2 and T3 were extremely significantly higher than CON (*p* < 0.01). IL-10 levels were extremely significantly higher in T1, T2, and T3 than in CON (*p* < 0.01). For pro-inflammatory factors, IFN-γ levels in both T2 and T3 were significantly lower than those in CON (*p* < 0.01). IL-1β levels in both T2 and T3 were significantly lower than those in CON (*p* < 0.01). IL-6 levels in T1, T2, and T3 were all significantly lower than those in CON (*p* < 0.01). IL-8 levels in T1, T2, and T3 were all significantly lower than those in CON (*p* < 0.01). For IL-18, T3 was significantly lower than CON (*p* < 0.01). For TNF-α, T1 and T3 was significantly lower than CON (*p* < 0.05).

Regarding plasma lipid metabolism, TC levels in T3 were significantly higher than those in CON (*p* < 0.05). GLU levels in both T2 and T3 were significantly higher than those in CON (*p* < 0.05). There were no significant differences in LAC or TG between any of the experimental groups and CON (*p* > 0.05).

Regarding plasma enzyme activity, AST levels were significantly higher in both T2 and T3 compared to CON (*p* < 0.05). γ-GT levels were significantly higher in all experimental groups compared to CON (*p* < 0.05). LDH levels were extremely significantly lower in T1 than in CON (*p* < 0.01), and significantly lower in both T2 and T3 than in CON (*p* < 0.05). There were no significant differences in ALT, alkaline ALP, γ-GT levels or CK levels among the groups (*p* > 0.05).

### 3.4. A Non-Targeted Metabolomics Analysis of the Effects of Sodium Butyrate on Blood Metabolism in Tumbler Pigeons After Competition

#### 3.4.1. Correlation Analysis of QC Samples Based on Relative Quantification of Metabolites

**Figure 2:**
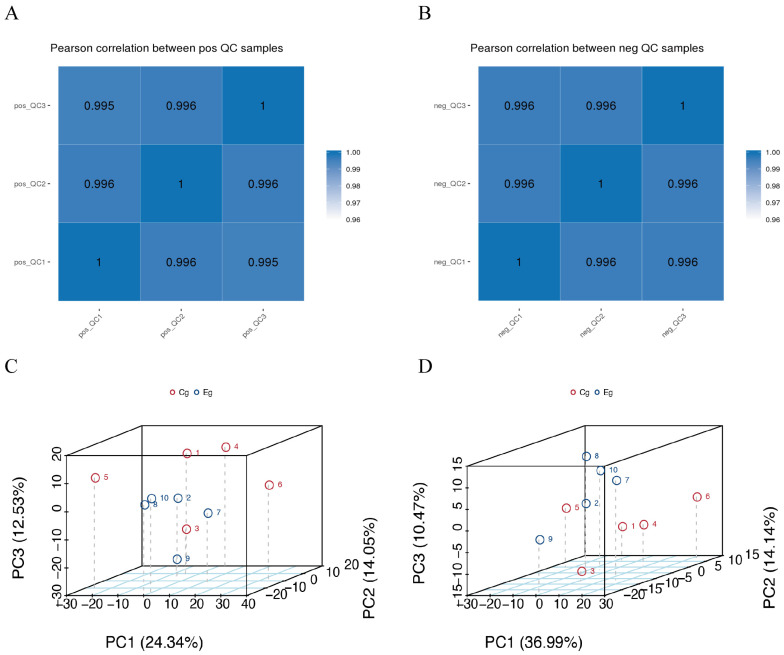
Quality control (QC) assessment and principal component analysis (PCA) of untargeted metabolomics data from tumbler pigeon plasma samples. (**A**) Pearson correlation analysis of QC samples in positive ion mode (pos); (**B**) Pearson correlation analysis of QC samples in negative ion mode (neg); (**C**) 3D PCA score plot of the first three principal components in positive ion mode; (**D**) 3D PCA score plot of the first three principal components in negative ion mode. In panels (**C**) and (**D**), red circles represent the control group (Cg), and blue circles represent the experimental group (Eg). The percentages on each principal component axis indicate the proportion of explained variance.

Plasma samples from CON and T2 were selected for untargeted metabolomics analysis. Analysis of QC Sample Correlation (Figure 2A,B): The correlation coefficients for QC samples in both positive and negative ion modes were above 0.99. This indicates that the LC-MS-based metabolite detection process in this study exhibits good stability and high data quality, meeting the requirements for subsequent research data. The QC samples cluster closely together in both positive and negative ion modes (Figure 2C,D), with minimal variation among samples, indicating that the detection method used in this study is stable and produces high-quality data.

#### 3.4.2. The Impact of Differential Metabolites in Plasma

**Figure 3:**
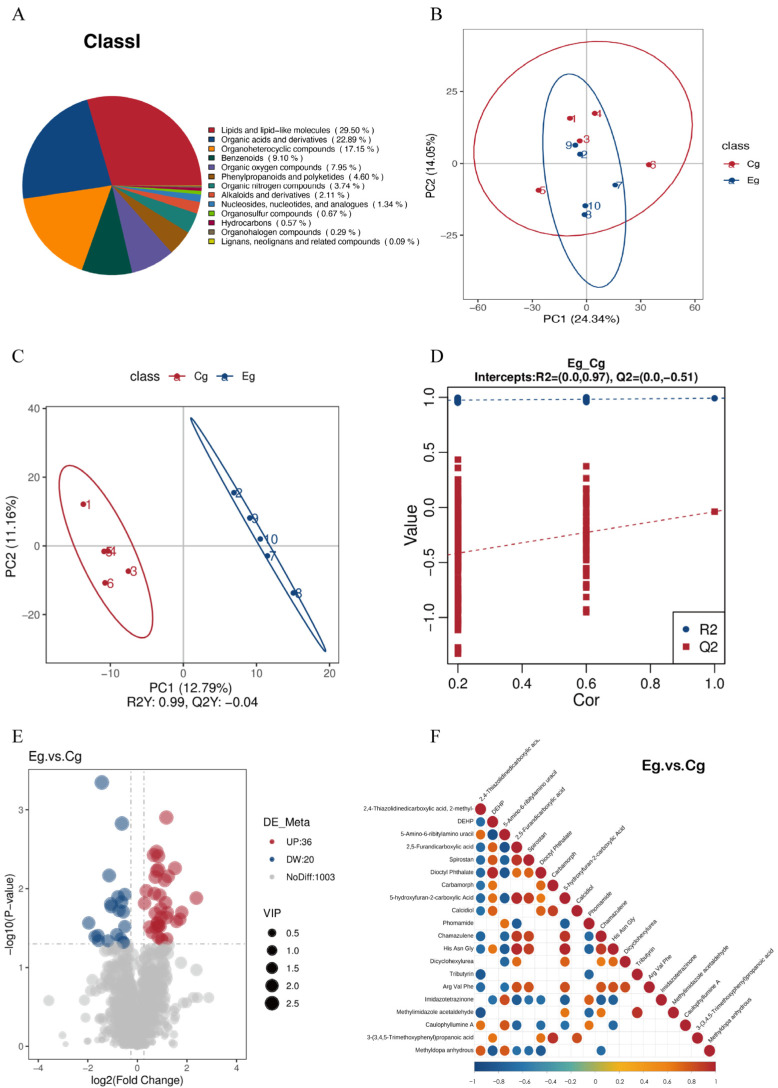
Untargeted metabolomic analysis of tumbler pigeon plasma in positive ion mode. (**A**) Chemical classification of annotated metabolites; (**B**) Unsupervised PCA score plot of control (Cg, red) and sodium butyrate-treated experimental (Eg, blue) groups; (**C**) Supervised PLS-DA score plot between groups; (**D**) PLS-DA permutation validation plot; (**E**) Volcano plot of differentially expressed metabolites (DEMs); (**F**) Heatmap of the top 25 DEMs.

As shown in Figure 3, in positive ion mode, metabolites were classified into 13 categories (Figure 3A), primarily involving lipids and lipid-like molecules (29.50%), organic acids and derivatives (22.89%), organoheterocyclic compounds (17.15%), benzenoids (9.10%), organic oxygen compounds (7.95%), phenylpropanoids and polyketides (4.60%), organic nitrogen compounds (3.74%), Alkaloids and derivatives (2.11%), Nucleosides, nucleotides, and analogues (1.34%), Organosulfur compounds (0.67%), Hydrocarbons (0.57%), Organohalogen compounds (0.29%), Lignans, neolignans, and related compounds (0.10%).

**Figure 4:**
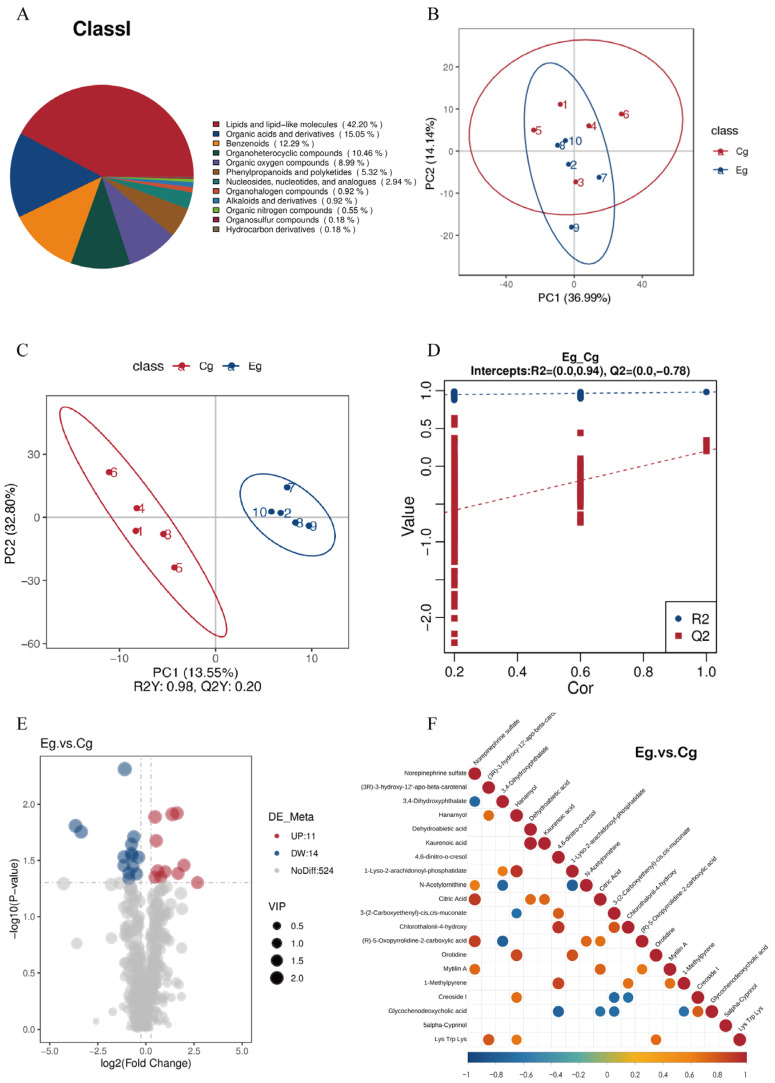
Untargeted metabolomic analysis of tumbler pigeon plasma in negative ion mode. (**A**) Chemical classification of annotated metabolites; (**B**) Unsupervised PCA score plot of control (Cg, red) and sodium butyrate-treated experimental (Eg, blue) groups; (**C**) Supervised PLS-DA score plot between groups; (**D**) PLS-DA permutation validation plot; (**E**) Volcano plot of differentially expressed metabolites (DEMs); (**F**) Heatmap of the top 25 DEMs.

Principal component analysis revealed that the control group and experimental group models (Figure 3B) contributed 36.99% to PC1 and 14.14% to PC2. Plasma metabolites were analyzed using PLS-DA (Figure 3C and Figure 4D) to identify potential biomarkers. In all models, the R^2^ value was greater than the Q^2^ value, and the intercept of the Q^2^ regression line with the *Y*-axis was less than 0, indicating that the models were reliable and not overfitted. A threshold of VIP > 1.0 was set, FC > 1.2 or FC < 0.833 and *p*-value < 0.05. The results of the differential metabolite screening are shown in (Figure 3E). Analysis of the control and experimental groups revealed 56 differential metabolites, including 36 upregulated and 20 downregulated metabolites. The heatmap of differential metabolites (Figure 3F) revealed distinct abundance patterns between the control and experimental groups, indicating that sodium butyrate supplementation altered the plasma metabolic profile.

As shown in Figure 4, in negative ion mode, metabolites were classified into 12 categories (Figure 4A), primarily involving lipids and lipid-like molecules (42.20%), organic acids and derivatives (15.05%), benzenoids (12.29%), organoheterocyclic compounds (10.46%), organic oxygen compounds (8.99%), phenylpropanoids and polyketides (5.32%), nucleosides, nucleotides, and analogues (2.94%), Organohalogen compounds (0.92%), Alkaloids and derivatives (0.92%), Organic nitrogen compounds (0.55%), Organosulfur compounds (0.18%), and Hydrocarbon derivatives (0.18%).

Principal component analysis revealed that the control group and experimental group models (Figure 4B) contributed 24.34% to PC1 and 14.05% to PC2. Plasma metabolites were analyzed using PLS-DA models (Figure 4C,D). In all models, the R^2^ values were greater than the Q^2^ values, and the intercepts of the Q^2^ regression lines with the *Y*-axis were less than 0, indicating that the models were reliable and not overfitted. A threshold of VIP > 1.0 was set, FC > 1.2 or FC < 0.833 and *p*-value < 0.05. The results of the screening for differentially expressed metabolites are shown in (Figure 4E). Analysis of the control group versus Group II revealed 25 differentially expressed metabolites, with 11 upregulated and 14 downregulated. The heatmap of differential metabolites (Figure 4F) revealed distinct abundance patterns between the control and experimental groups, indicating that sodium butyrate supplementation altered the plasma metabolic profile.

#### 3.4.3. KEGG Classification and Enrichment Analysis of Differentially Expressed Metabolites

KEGG classification analysis showed that most differential metabolites identified in both positive and negative ion modes were associated with metabolic processes. In the positive ion mode, differential metabolites were mainly involved in lipid metabolism, amino acid metabolism, and the metabolism of cofactors and vitamins. In the negative ion mode, differential metabolites were primarily associated with amino acid, lipid, carbohydrate, and nucleotide metabolism, indicating that sodium butyrate supplementation influenced multiple aspects of systemic metabolism.

**Figure 5:**
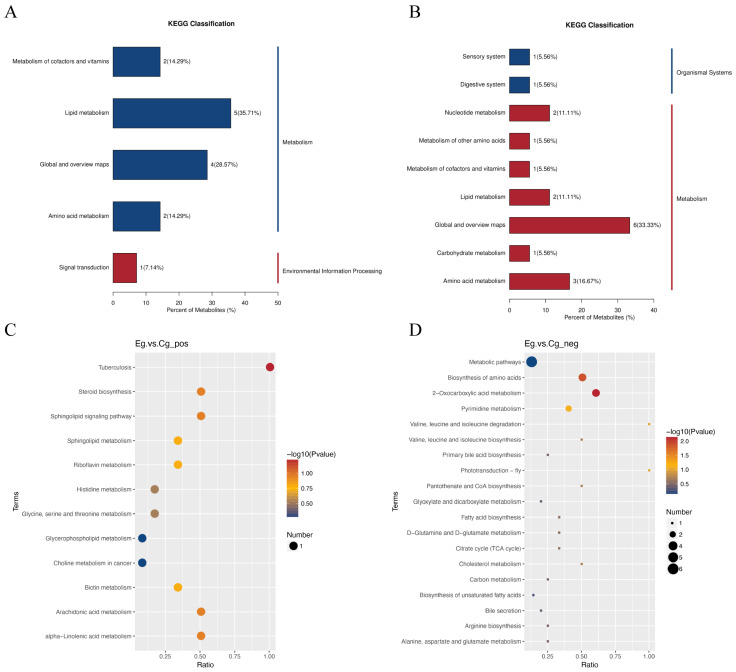
KEGG pathway enrichment analysis of differentially expressed metabolites between CON and T2. (**A**) KEGG classification of DEMs in positive ion mode; (**B**) KEGG classification of DEMs in negative ion mode; (**C**) Enrichment bubble plot of DEMs in positive ion mode; (**D**) Enrichment bubble plot of DEMs in negative ion mode.

KEGG enrichment analysis further revealed several significantly enriched pathways (Figure 5). In the positive ion mode, arachidonic acid metabolism, α-linolenic acid metabolism, steroid biosynthesis, and sphingolipid signaling pathways were among the most enriched pathways. In the negative ion mode, 2-oxocarboxylic acid metabolism, biosynthesis of amino acids, and pyrimidine metabolism were significantly enriched. These pathways are closely related to inflammatory regulation, lipid metabolism, amino acid turnover, and energy metabolism, suggesting that sodium butyrate supplementation may modulate physiological homeostasis through coordinated metabolic adaptations.

#### 3.4.4. Gas and Biochemical Parameters in Tumbler Pigeons

**Figure 6:**
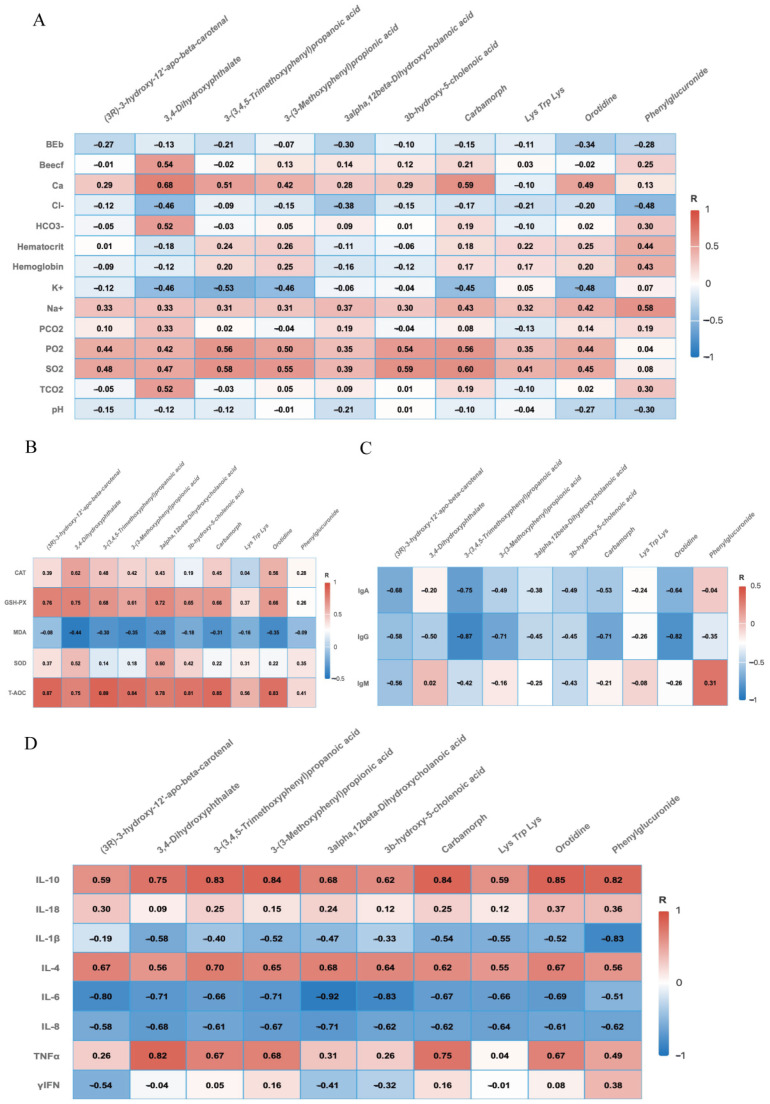
Correlation heatmaps of plasma differentially expressed metabolites with physiological parameters in tumbler pigeons. (**A**) Blood gas parameters; (**B**) Antioxidant indices; (**C**) Immunoglobulin levels; (**D**) Inflammatory cytokines. The color scale represents the Pearson correlation coefficient (R), with red for positive correlation and blue for negative correlation.

Based on the criteria of VIP > 1.8, *p* < 0.05, and FC ≥ 2, we identified five significantly up- and downregulated metabolites between the CON and T2 groups in both positive and negative ion modes (Table S3). We then performed correlation analyses between these differentially expressed metabolites and blood gas parameters as well as various blood biochemical indicators. The results are shown in Figure 6. Spearman’s r analysis revealed significant correlations between blood gas parameters, antioxidant and immune markers, inflammatory factors, and plasma non-targeted differentially expressed metabolites.

Blood gas parameters primarily reflect the acid-base balance and oxygen transport capacity of pigeons after intense flight. As shown in Figure 5A, the metabolite hydroxyectoine was strongly positively correlated with Cl^−^ (R = 0.81) in the positive ion mode. In the anion mode, the metabolite Mytilin A showed a negative correlation with PvO_2_ and SvO_2_; however, the biological significance of this association remains unclear and warrants further investigation.

Antioxidant and protein-based immunological markers reflect the body’s oxidative stress and defense levels. GSH-PX, SOD, and T-AOC are key defense systems that scavenge free radicals and prevent cellular damage; MDA is a product of lipid peroxidation that reflects the extent of cellular damage. Analysis of the correlation between differential metabolites and antioxidant markers in plasma, as shown in Figure 5B, reveals that under the negative ion mode, GSH-PX exhibits a strong negative correlation with norepinephrine sulfate (R = −0.85) and Lys Trp Lys (R = −0.90), while showing a strong positive correlation with (3R)-3-hydroxy-12′-apo-β-carotenal (R = 0.76). T-AOC showed a highly negative correlation with Lys Trp Lys (R = −0.89).

IgA, IgG, and IgM are the core immunoglobulins. Analysis of the correlation between differential metabolites in plasma and immune markers, as shown in Figure 5C, reveals that, regarding the immune system, IgG correlates with phenylglucuronide (R = −0.82) in anion mode, and in cation mode with 3-(3,4,5-trimethoxyphenyl)propanoic acid (R = −0.87).

Exercise-induced microdamage triggers an inflammatory response. IL-6 and IL-1β are pro-inflammatory factors involved in initiating the inflammatory response; while IL-10 and IL-4 are typical anti-inflammatory factors that help limit inflammation and promote muscle and tissue repair. Analysis of the correlation between plasma differential metabolites and inflammatory factors, as shown in Figure 5D, reveals that IL-6 exhibits a very strong positive correlation with chlorothalonil-4-hydroxy (R = 0.90) in anion mode (R = 0.90) in negative ion mode, and showed a very strong negative correlation with 3β-hydroxy-5-cholenoic acid (R = −0.92) in positive ion mode. The anti-inflammatory factor IL-10 showed a high positive correlation with orotidine (R = 0.85) and phenylglucuronide (R = 0.82) in the anion mode. However, because the biological relevance of these metabolites to sodium butyrate supplementation is uncertain, this association should be interpreted with caution.

## 4. Discussion

### 4.1. The Effect of Sodium Butyrate Supplementation on Apparent Nutrient Metabolism in Tumbler Pigeons

The apparent metabolic rate of nutrients in the diet directly reflects an animal’s ability to utilize them and is one of the core indicators for evaluating the nutritional value of feed [30]. However, both diet type and formulation [31] influence the apparent digestion and metabolism of feed nutrients, while the animal’s own intestinal structure and morphology [32] and the gut microbiota [33] are also significant influencing factors. Research by Gorenz, B. et al. [34] indicates that adding heat-stable xylanase to wheat-based pelleted feed can improve growth performance in broiler chickens, while simultaneously reducing bolus viscosity and increasing the metabolizable energy content of the diet.

However, the dietary composition of tumbler pigeons differs significantly from that of traditional broilers. During daily feeding and high-intensity tumbler training, tumbler pigeons typically consume whole grains such as corn, sorghum, and peas as their staple diet. Although this whole-grain feeding regimen aligns with pigeons’ natural foraging and gizzard grinding habits, the hard plant pericarp of these grains is rich in insoluble fiber, which significantly reduces the rate of nutrient digestion and metabolism [35]. Building on this nutritional context, Liu, J.J. et al. [36] investigated how adding 0.025 g/kg of a Chinese herbal preparation to the diet can improve the growth performance, serum antioxidant capacity, and intestinal digestive enzyme activity of meat pigeons; similarly, Liang, Y.Y. et al. [37] found that different selenium sources can enhance the immune performance and antioxidant function of squab and regulate intestinal morphological development. These studies collectively demonstrate that improving pigeon intestinal morphology and increasing gut microbiota abundance through exogenous additives can significantly enhance dietary nutrient digestion and metabolism. As a short-chain fatty acid salt, sodium butyrate serves as the primary direct energy source for the rapid renewal and proliferation of intestinal mucosal epithelial cells; it effectively promotes intestinal villus development, increases the absorptive surface area, and plays a significant role in regulating intestinal microecological health [38]. In this study, we found that supplementing rock pigeons with medium-to-high doses of sodium butyrate significantly improved the digestibility of DM in rock pigeons. This is consistent with the findings of Sadurní, M. [39] and others. An appropriate dose of sodium butyrate can strengthen the intestinal barrier in broiler chickens, thereby improving the digestibility and utilization of dry matter and organic matter in the diet. This indicates that supplementing with an appropriate level of sodium butyrate improves rock pigeons’ digestive capacity to absorb essential nutrients in the diet. Under the conditions of this study, sodium butyrate had no significant effect on the apparent metabolic rate of OM, which may be related to the high basal diet digestibility in rock pigeons and to the birds being in a stable training state throughout the experimental period [40]. Regarding energy metabolism, the DE in Treatment T3 was significantly higher than that in the control group and Treatment T1. This is consistent with the findings of Zhen, L. et al. [41], who reported that sodium butyrate can improve the availability of dietary energy by enhancing the intestinal microenvironment, promoting fatty acid absorption, and regulating energy metabolism-related pathways, indicating that supplementing with a certain dose of sodium butyrate can improve the intestinal morphology of livestock and poultry, thereby increasing the efficiency of dietary energy utilization. For tumbler pigeons, the increase in ME may contribute to a greater availability of dietary energy during periods of intensive flight training. Under the conditions of this experiment, supplementing the basal diet with 12 mg/d and 18 mg/d of sodium butyrate can, to a certain extent, improve the digestion and utilization of dietary nutrients in high-exercise-load tumbler pigeons. This regulatory effect, characterized by the maintenance and optimization of digestive and metabolic status, may contribute to improved nutrient utilization and physiological adaptation during periods of increased exercise demand [42].

### 4.2. The Effects of Sodium Butyrate Supplementation on Blood Parameters in Tumbler Pigeons

Blood gas analysis in competition animals is a key indicator for assessing exercise intensity, metabolic load, and physiological adaptability, providing a dynamic reflection of respiratory function, acid-base balance regulation mechanisms, and the efficiency of oxygen supply and utilization under exercise stress [43].

During high-intensity tumbler flights, tumbler pigeons experience significant physiological stress from fatigue-inducing exercise; changes in blood acid-base balance are a key indicator of their exercise metabolism and recovery capacity [44]. Liu, X.X. et al. [45] found that under normal conditions, there is no significant change in plasma pH after exercise in tumbler pigeons. The results of this study showed similar patterns in the low- and high-dose groups, with no significant differences before and after the simulated race. However, in the intermediate-dose group, both the pre-race pH and BEb values were significantly higher than those in the control and high-dose groups. Furthermore, after the simulated race, the BEb levels in the intermediate-dose group decreased significantly, while maintaining the body’s pH balance. This suggests that supplementing with a certain dose of sodium butyrate may lead to an increase in the blood’s alkaline reserve in tumbler pigeons prior to exercise, thereby raising blood pH. pH and BEb are core indicators for measuring blood acid-base balance [46], with BEb directly reflecting the degree of non-respiratory acid-base imbalance in whole blood; its elevation typically indicates a tendency toward metabolic alkalosis or an enhancement of compensatory alkaline reserves [47]. Results similar to those of this study have been observed in other ruminants. Liu, J.J. et al. [48] found that supplementing Ili horses with plant polyphenols increased plasma intracellular base excess, total carbon dioxide, hemoglobin content, and pH. It is hypothesized that butyric acid, as the active component of sodium butyrate, enters cells and primarily provides energy through β-oxidation in mitochondria; this metabolic process generates HCO_3_^−^, which may exert a mild alkalizing effect [49]. Pre-race pH values in the intermediate-dose group were significantly higher than those in the control and high-dose groups, and also significantly higher than those in the intermediate-dose group post-race; however, there were no significant differences in pH values among the groups post-race. These results indicate that a moderate dose of sodium butyrate supplementation caused an increase in blood pH in pigeons before the race, resulting in an alkaline shift. This may be related to sodium butyrate enhancing colonic bicarbonate secretion and systemic buffering capacity. Squires, E.J. et al. [50] found in a broiler chicken study that butyrate can stimulate NBCe1 expression and renal HCO_3_^−^ reabsorption. Butyrate promotes bicarbonate production and absorption by enhancing the exchange of Cl^−^ and HCO_3_^−^ in the intestine, thereby increasing blood buffering capacity [51]. The alkaline shift observed in the moderate-dose group before the race may help buffer exercise-induced acid-base disturbances and support physiological homeostasis during exercise [52]. However, there were no significant differences in pH post-competition, suggesting that sodium butyrate may maintain homeostasis by accelerating CO_2_ elimination via the lungs and acid excretion via the kidneys [53], thereby aiding the body in rapidly restoring acid-base balance after intense exercise. The fact that the concentration of BE b in the intermediate-dose group was also significantly higher than in the other groups further supports this view. This mechanism of action may involve multiple pathways, including sodium butyrate’s ability to improve gut health, regulate microbial metabolism, increase bicarbonate production, and promote renal bicarbonate reabsorption. As a short-chain fatty acid salt, sodium butyrate is considered a potential antibiotic alternative and growth promoter in animal production [54,55]. It is absorbed in the intestine and enters the liver via the portal vein; some butyrate may be converted into bicarbonate, thereby increasing the blood’s alkaline reserve [56]. Additionally, sodium butyrate may enhance renal tubular reabsorption of bicarbonate by regulating anion transporters, thereby improving the blood’s buffering capacity. Furthermore, with the exception of the intermediate dose group, there were no significant differences in pre- and post-race pH, BE ecf, BE b, and HCO_3_^−^ levels among the experimental groups, indicating that supplementing with a certain dose of sodium butyrate does not adversely affect the acid-base balance of tumbler pigeons after training flights. Immediate measurements following a single exercise session are insufficient to detect significant differences in pH between the experimental and control groups; further validation is required in higher-intensity exercise stress models.

During exercise in tumbler pigeons, gas exchange plays an important role in maintaining physiological function and metabolic homeostasis. Indicators such as partial pressure of PCO_2_, TCO_2_, PO_2_, and SO_2_ typically remain relatively stable; together, they reflect the functional status of tissue and pulmonary gas exchange within the body [57]. Monitoring these physiological parameters is crucial for assessing the health and exercise performance of pigeons. Liu, X.X. et al. [45] found that under normal conditions, plasma PvCO_2_ and TCO_2_ concentrations significantly decrease after exercise in tumbler pigeons. Contrary to these findings, the results of this study indicate that supplementing with a moderate dose of sodium butyrate effectively controls the rise in PvCO_2_ levels, suggesting that sodium butyrate supplementation may be associated with altered carbon dioxide handling during exercise. Furthermore, the post-race PvO_2_ levels in the moderate-dose group were significantly higher than pre-race levels, suggesting that supplementation with a certain dose of sodium butyrate may be associated with changes in oxygen availability and transport status, thereby increasing the arterial blood oxygen content in tumbler pigeons. There were no significant differences in gas exchange parameters such as PvCO_2_, PvO_2_, TCO_2_, and SvO_2_ among the groups post-race. This is consistent with the findings of Liu, J.J. et al. [58] in their study of Yili horses, where supplementation with mangiferin had no significant effect on plasma electrolyte levels, acid-base balance, or blood gas parameters. This suggests that the physiological state of the tumbler pigeons recovered after the race, the immediate effects of sodium butyrate diminished, or the post-race recovery mechanisms masked the long-term effects of sodium butyrate. It is speculated that the significant pre-race differences may be related to sodium butyrate’s regulation of the gut microbiota, improvements in nutrient absorption and energy metabolism, and subsequent effects on respiratory and circulatory system functions. The absence of significant post-race differences may be related to physiological adjustments and metabolic recovery in tumbler pigeons.

Hematocrit (Hct) and hemoglobin (Hb) are important hematological indicators for assessing the body’s oxygen-carrying capacity and protein nutritional status [59]. Hb is the primary iron-containing protein in red blood cells; its core physiological function lies in reversibly binding and transporting oxygen to peripheral tissues, while also assisting in the transport of a portion of metabolic carbon dioxide back to the lungs for excretion. Therefore, Hb levels directly reflect the blood’s oxygen-carrying reserve capacity. Hct is defined as the percentage of whole-blood volume occupied by red blood cells and is a crucial indicator of dehydration or anemia. Hct is not only a key determinant of tissue oxygenation efficiency but also a primary factor influencing blood viscosity [60]. Liu, X.X. et al. [45] found that under normal conditions, plasma Hct and Hb concentrations decrease significantly after exercise in tumbler pigeons. The results of this study, however, contradict this finding: Hb and Hct concentrations across all groups rapidly returned to pre-race resting levels after the race, and Hb levels in the high-dose group significantly increased post-race. This indicates that supplementing with a certain dose of sodium butyrate may enhance metabolic activity and oxygen transport capacity, which could be beneficial for physiological adaptation to exercise. Furthermore, the higher Hb concentration observed in the high-dose group may indicate an increased oxygen-carrying capacity, although direct measurements of exercise performance were not conducted in the present study.

Electrolytes are essential minerals that maintain vital functions such as normal cellular function, nerve conduction, muscle contraction, and bone health [61]. Ions such as Na^+^, K^+^, Ca^2+^, and Cl^−^ play key roles in maintaining osmotic and acid-base balance, as well as neuromuscular excitability, between the intracellular and extracellular spaces [62]. Na^+^ is the primary cation in extracellular fluid, and its concentration is a key determinant of plasma osmolarity. Serum sodium concentration is primarily determined by the ratio of total exchangeable solutes to total body water. Therefore, changes in serum sodium concentration primarily reflect fluid balance rather than changes in total body sodium content. The significant post-race increase in Na^+^ levels observed in the group supplemented with an intermediate dose of sodium butyrate is consistent with the earlier analysis suggesting that this group may exhibit dehydration symptoms following the pigeon race. Ca^2+^ interacts with ion channels and receptors on the cell membrane to regulate neuromuscular excitability [63]. When acidosis occurs in the body, the concentration of H+ in the blood rises. These hydrogen ions bind to albumin, thereby reducing the binding sites for calcium ions on albumin, leading to the release of more free calcium ions into the blood and an increase in plasma calcium ion concentration [64]. In this study, Ca^2+^ levels remained stable in all experimental groups after the race, with the exception of the control group, indicating that supplementing with a specific dose of sodium butyrate can effectively alleviate the acidosis that may occur in racing pigeons following high-intensity exercise. Exercise-induced acidosis is a common physiological phenomenon; for example, blood pH decreases after intense exercise. Endurance racehorses also exhibit changes in water, electrolyte, and acid-base parameters during competition [65]. Consistent with the findings of this study, Senda, M. et al. [66] found that serum calcium levels may rise following intense exercise, which is associated with increased bone resorption. In the racing pigeon test group, no significant alterations in K^+^ or Cl^−^ concentrations were observed before and after the race, indicating that sodium butyrate supplementation did not disrupt electrolyte homeostasis under the conditions of the present study. However, considering that changes in AST in certain biochemical indicators, were observed in some treatment groups, further studies are required to comprehensively evaluate the long-term physiological safety of sodium butyrate supplementation in tumbler pigeons. Endurance racehorses also exhibit alterations in water, electrolyte, and acid–base parameters during competition. Previous studies have reported that serum calcium concentrations may increase following intense exercise, potentially in association with changes in bone metabolism. In the present study, no significant changes in K^+^ or Cl^−^ concentrations were observed before and after the race in the sodium butyrate-supplemented groups, suggesting that electrolyte homeostasis was generally maintained under the experimental conditions. Although the underlying mechanisms remain unclear, the reported effects of sodium butyrate on intestinal physiology, including improvements in intestinal morphology and microbial composition, may contribute to mineral utilization and metabolic regulation [67].

Among the tested supplementation levels, 12 mg/d sodium butyrate was associated with the most favorable overall response in several blood gas parameters. These findings suggest that sodium butyrate supplementation may be associated with the maintenance of acid–base balance and physiological homeostasis during periods of exercise challenge. However, because most blood gas variables remained within normal physiological ranges and several significant differences were relatively small in magnitude, the biological relevance of these changes should be interpreted cautiously.

### 4.3. The Effects of Sodium Butyrate Supplementation on Plasma Biochemical Parameters in Tumbler Pigeons

#### 4.3.1. Physiological Interpretation of Changes in Routine Plasma Biochemical Parameters

Routine plasma biochemical parameters are key indicators for assessing an animal’s physiological state, metabolic function, and health status. Parameters such as TP, ALB, GLB, A/G, and UA collectively reflect protein metabolism, hepatic protein metabolism, and the excretion of nitrogenous waste products in pigeons. TP reflects the body’s overall protein nutritional reserves and anabolic metabolism; ALB is primarily synthesized by the liver and serves as a crucial indicator of hepatic synthetic function, nutritional status, and vascular colloid osmotic pressure. In this study, ALB was significantly reduced in the low-dose group. Contrary to these findings, He Liu, H.K. et al. [68] observed that the addition of sodium butyrate had no significant effect on serum TP in Hu sheep, whereas ALB initially increased and then decreased with increasing sodium butyrate supplementation. It is speculated that the findings of this study are more likely attributable to physiological adaptive redistribution mechanisms during the early stages of supplementation. As the primary energy substrate for colonic epithelial cells, sodium butyrate can rapidly enhance the expression of tight junction proteins, promote goblet cell differentiation and mucus secretion, thereby facilitating intestinal mucosal repair [69,70]. During this process, the body may prioritize allocating limited amino acid resources to the rapid repair and reconstruction of damaged intestinal mucosa or to the synthesis of immunoglobulins [71]. This leads to a relative shortage of amino acid precursors for albumin synthesis in the liver, resulting in a transient decrease in circulating ALB and TP. As the sodium butyrate dose increased, TP and ALB levels in the high-dose group recovered or approached those of the control group. This is speculated to be because, as the sodium butyrate dose increased, its ability to promote nutrient absorption efficiency gradually became dominant, thereby maintaining homeostasis in protein metabolism. Sodium butyrate enhances absorption by upregulating the expression of intestinal nutrient transporters. Among these, sodium–glucose cotransporter 1 (SGLT1) and peptide transporter 1 (PepT1) are two key transporters. SGLT1 is primarily responsible for glucose absorption, while PepT1 mediates the absorption of dipeptides and tripeptides; these short peptides are hydrolyzed in the intestine, releasing amino acids for animal utilization. Sodium butyrate improves the morphology of intestinal microvilli and enhances amino acid absorption and utilization by upregulating transporters such as SGLT1 and PepT1 [72]. This improvement in intestinal function enables the body to absorb nutrients more efficiently, thereby providing sufficient amino acids for hepatic protein synthesis and allowing ALB and TP levels to rebound. There were no significant differences in GLB, A/G, and UA among the groups, indicating that sodium butyrate supplementation within the tested dosage range did not adversely affect the pigeons’ basal protein metabolic balance, hepatic synthetic function, or purine metabolism, and their overall metabolic status remained well maintained.

#### 4.3.2. The Effect of Sodium Butyrate Supplementation on Plasma Antioxidant Parameters in Tumbler Pigeons

The antioxidant system plays a crucial role in livestock and poultry by helping the body resist oxidative stress damage, maintain cellular integrity, and delay fatigue. MDA is one of the primary end products of lipid peroxidation and serves as a classic biomarker for assessing oxidative stress-induced damage to cell membrane lipids [73]. SOD, as an important antioxidant enzyme, catalyzes the superoxide anion radicals generated by cellular respiration, converting them into relatively less toxic hydrogen peroxide through a dismutation reaction [74], while CAT decomposes the hydrogen peroxide produced by SOD into harmless water and oxygen [75]; GSH-Px is another important family of antioxidant enzymes that utilizes reduced glutathione to reduce hydrogen peroxide or organic peroxides into water or corresponding alcohols, while T-AOC reflects the body’s overall antioxidant defense level [76]. Extensive research has confirmed that sodium butyrate can significantly enhance the antioxidant capacity of livestock and poultry. Sun, H.X. et al. [77] found that adding sodium butyrate to the diet significantly increased the levels of SOD, CAT, GSH-Px, and T-AOC in the serum of squab, while simultaneously reducing MDA content. Zhang, X. [78] et al. found that adding coated sodium butyrate to the diet significantly increased T-AOC and GSH-Px activity in the serum of 42-day-old broiler chickens. The results of this study are consistent with these findings. Compared to the control group, the medium- and high-dose groups exhibited significant increases in SOD, CAT, GSH-PX, and T-AOC levels. These findings suggest that dietary sodium butyrate supplementation was associated with enhanced antioxidant enzyme activities in tumbler pigeons. The metabolomic enrichment of lipid-related pathways, particularly arachidonic acid metabolism and sphingolipid signaling, may partly explain these antioxidant responses, although direct causal relationships remain to be established. Improved antioxidant status may contribute to the maintenance of physiological homeostasis and support exercise-related metabolic adaptation. Under the conditions of the present study, supplementation with 18 mg/d sodium butyrate was associated with higher antioxidant enzyme activities, suggesting a beneficial role in maintaining physiological function during periods of increased exercise demand.

#### 4.3.3. Effects of Sodium Butyrate Supplementation on Plasma Immune Parameters in Rock Doves

Immunoglobulins are key effector molecules in the body’s humoral immune response and are primarily classified into three major categories: IgA, IgM, and IgG. IgA serves as the first line of immune defense on mucosal surfaces, exerting local immune protection by blocking pathogen adhesion, neutralizing toxins, and regulating microbial homeostasis. IgM is the first antibody produced during the primary immune response; it possesses a strong ability to activate complement and exhibits high-affinity multivalent binding properties [79]. In contrast, IgG is the most abundant immunoglobulin in serum and plays a leading role in systemic immune protection and immune memory [80]. Multiple studies have also shown that sodium butyrate plays a significant role in enhancing the immune capacity of livestock and poultry. For example, Liu, L.H. [81] found that sodium butyrate can enhance the immune function of broiler chickens by increasing intestinal mucosal S-IgA levels. Similarly, Li, D.D. et al. [82] found that adding sodium butyrate to the diet promotes the development of immune organs in weaned piglets, increases serum immunoglobulin levels and the secretion of secretory IgA in the small intestinal mucosa, and enhances the immune function of weaned piglets. Zhao, H.L. et al. [83] found that sodium butyrate can increase the immune organ index in animals, thereby promoting an increase in serum immunoglobulins in calves. In this study, compared with the control group, all immunoglobulin levels were elevated in the high-dose group, suggesting that supplementation with 18 mg/d sodium butyrate may support humoral immune status in tumbler pigeons.

#### 4.3.4. Effects of Sodium Butyrate Supplementation on Plasma Inflammatory Markers in Rock Doves

The inflammatory response helps the body fight infections and injuries. Moderate inflammation is necessary for defense, but excessive or persistent inflammation can lead to tissue damage, metabolic disorders, and immunosuppression [84]. IL-4 is primarily secreted by Th2 cells and mast cells. It promotes B-cell class switching, induces IgE production, and suppresses Th1-type pro-inflammatory responses [85]. IL-10 is a key broad-spectrum anti-inflammatory cytokine that significantly downregulates various pro-inflammatory factors by inhibiting the NF-κB and MAPK signaling pathways [86]. IFN-γ is a potent pro-inflammatory factor that exerts its anti-intracellular pathogen effects by activating macrophages, enhancing antigen presentation, and promoting Th1 cell differentiation [87]. IL-1β is one of the core mediators of early-phase inflammation, strongly inducing fever, acute-phase protein synthesis, and leukocyte recruitment [88]; IL-6 triggers the liver to synthesize acute-phase proteins such as C-reactive protein, induces fever, and promotes B-cell and Th17 cell differentiation, playing a central role in the systemic effects of inflammation [89,90]. IL-8 is a chemokine whose primary function is to specifically recruit neutrophils to sites of inflammation [91]. TNF-α is a key initiator of the inflammatory cascade [92]. IL-18 exacerbates inflammatory damage [87]. The results of this study indicate that the levels of the anti-inflammatory factors IL-4 and IL-10 were significantly higher in the groups supplemented with different doses of sodium butyrate compared to the control group, with the high-dose group showing the best effect, which is consistent with the findings of Zhang, W.H. et al. [93]. In a broiler chicken model subjected to stressors under modern intensive farming conditions, the addition of an appropriate amount of sodium butyrate reduced serum concentrations of IL-6 and TNF-α in certain age groups. Zhang, X. et al. [94] also confirmed in a mouse model of necrotizing enteritis that sodium butyrate effectively reduced the levels of pro-inflammatory cytokines IL-1β, IL-6, and TNF-α in the intestine and serum, while increasing the anti-inflammatory cytokine IL-10. This indicates that sodium butyrate effectively promotes the production of cytokines that inhibit inflammation and promote tissue repair. Additionally, under the conditions of this experiment, the levels of pro-inflammatory factors γ-IFN, IL-1β, IL-6, IL-8, TNF-α, and IL-18 all decreased significantly. Furthermore, the extent of the increase in anti-inflammatory factors and the decrease in pro-inflammatory factors increased with higher doses of sodium butyrate, with the most pronounced effect observed in the highest-dose group. Collectively, these results suggest that sodium butyrate supplementation was associated with a shift toward an anti-inflammatory profile characterized by increased anti-inflammatory cytokines and reduced pro-inflammatory cytokines. This interpretation is further supported by the metabolomic enrichment of arachidonic acid metabolism and sphingolipid-related pathways, both of which are known to participate in inflammatory regulation. Nevertheless, further studies are required to determine whether these metabolomic alterations directly mediate the observed cytokine responses. Dietary supplementation with sodium butyrate improved antioxidant status and modulated inflammatory responses in tumbler pigeons undergoing regular training and competition. Among the tested supplementation levels, 18 mg/d sodium butyrate generally produced the most favorable physiological responses. These findings suggest that sodium butyrate may support exercise-related physiological adaptation; however, direct measurements of athletic performance and recovery outcomes are required to confirm these potential benefits.

#### 4.3.5. Effects of Sodium Butyrate Supplementation on Plasma Glucose and Lipid Metabolism in Rock Doves

In the daily training of tumbler pigeons, carbohydrates and lipids are the primary sources of energy, and the balance of their metabolic processes is crucial for maintaining the birds’ normal physiological functions. TC is the total cholesterol content of all lipoproteins in the blood, and its level is an important indicator for assessing lipid metabolism [95]. TG is the primary form of stored energy in the body, and its levels reflect fat synthesis and catabolism [96]. GLU is one of the core indicators of energy metabolism in animals. For tumbler animals such as tumbler pigeons, blood glucose levels not only reflect their current energy reserve status and the intensity of their stress response but are also closely related to the capacity for liver glycogen mobilization and insulin sensitivity [97]. LAC is the end product of glycolysis and serves as a key indicator of tissue hypoxia and energy metabolism in animals. Lactate levels directly reflect exercise intensity, energy expenditure patterns, and the body’s ability to adapt to and clear exercise-induced stress [98]. Li, X.B. et al. [40] found that different dietary nutrient levels can influence glucose and lipid metabolism in tumbler pigeons, with T2 increasing serum GLU and TC levels in tumbler pigeons. The results of this study are generally consistent with these findings, as sodium butyrate supplementation increased plasma GLU and TC concentrations in certain treatment groups. Sodium butyrate may influence hepatic glucose metabolism by acting as a substrate and signaling molecule for hepatic gluconeogenesis [99]. Under conditions of increased energy demand, the liver may enhance gluconeogenesis to maintain glucose homeostasis, which could partly explain the elevation in plasma GLU. In addition, sodium butyrate has been reported to modulate lipid metabolism and nutrient utilization, potentially contributing to changes in circulating cholesterol concentrations [100]. However, the observed increases in GLU and TC should not be interpreted exclusively as beneficial physiological responses. These alterations may reflect enhanced availability of energy substrates and metabolic adaptation to exercise demands, but they may also be associated with transient changes in hepatic metabolic activity or nutrient partitioning. Therefore, the biological significance of these responses should be interpreted cautiously. Interestingly, the untargeted metabolomic analysis further revealed significant enrichment of pathways related to steroid biosynthesis, arachidonic acid metabolism, alpha-linolenic acid metabolism, amino acid biosynthesis, and 2-oxocarboxylic acid metabolism. These pathway alterations suggest that sodium butyrate supplementation may influence both lipid and energy metabolism at the systemic level, providing a potential mechanistic basis for the observed changes in plasma biochemical parameters. Nevertheless, because the present study did not directly assess metabolic fluxes or enzyme activities within these pathways, further investigations are required to clarify the causal relationships between metabolomic alterations and changes in glucose and lipid metabolism.

#### 4.3.6. Effects of Sodium Butyrate Supplementation on Plasma Enzyme Activity in Tumbler Pigeons

As an important biomarker, plasma enzyme activity reflects an animal’s physiological state, health status, tissue damage, and stress response. For tumbler animals, changes in plasma enzyme activity have specific physiological and practical significance and can be used to assess training load and fatigue levels, diagnose diseases, and predict performance [101]. In tumbler pigeons, plasma ALT activity is considered a more liver-specific enzyme, and elevated levels primarily indicate hepatocyte damage; elevated AST activity may indicate liver and muscle damage and is more prevalent in mitochondria; plasma ALP activity may be related to bone metabolism, intestinal absorption function, or stress responses; elevated plasma γ-GT activity is a sensitive indicator of liver disease; particularly in certain drug-induced liver injuries, γ-GT exhibits higher specificity than AST [102]. Elevated plasma LDH activity is typically associated with muscle damage and enhanced anaerobic metabolism under hypoxic conditions [103]. CK activity is a sensitive indicator of muscle damage and exercise load [104]. If both AST and CK are significantly elevated simultaneously, the diagnosis is more likely to be muscle damage. Li, X.B. et al. [40] found that different dietary nutrient levels can lead to changes in plasma enzyme activity in tumbler pigeons, with the T4 diet causing a significant increase in AST levels. It should be noted that AST activities were elevated in certain sodium butyrate-supplemented groups. Although the enzymes are commonly used as indicators of hepatic function, moderate increases do not necessarily reflect pathological liver damage in athletic birds. AST may also originate from skeletal muscle tissue, particularly under conditions of intensive exercise. Therefore, the observed increases may reflect enhanced metabolic activity associated with exercise adaptation and nutrient utilization. Nevertheless, because these markers can also be associated with altered hepatic workload, their biological significance should be interpreted cautiously. Additional studies incorporating liver histology and functional assessments are required to clarify the underlying mechanisms. At the same time, under the conditions of this study, all experimental groups showed a significant reduction in LDH activity, indicating that sodium butyrate supplementation was associated with lower plasma LDH activity, which may reflect reduced cellular leakage or altered metabolic responses, thereby reducing the release of LDH from cells into the bloodstream [105]. Li et al. [106] reported that sodium butyrate exerted a hepatoprotective effect against hepatic inflammatory injury in subchronic fluoride-exposed mice. In this study, sodium butyrate supplementation had no significant effect on post-race plasma ALT, ALP, γ-GT, or CK levels in tumbler pigeons, indicating no obvious alterations in these biochemical indicators following supplementation. Nevertheless, because AST activities increased in certain treatment groups, additional studies are needed to further evaluate the physiological relevance of these changes and the long-term safety of sodium butyrate supplementation.

### 4.4. Analysis of Non-Target Metabolite Profiles in the Plasma of Tumbler Pigeons Following Sodium Butyrate Supplementation

This study systematically revealed the broad effects of dietary sodium butyrate supplementation on the plasma metabolome of tumbler pigeons using untargeted metabolomics combined with KEGG pathway enrichment analysis. KEGG enrichment analysis of plasma metabolites revealed that, in the anion mode, differentially expressed metabolites were primarily enriched in the two major KEGG functional categories of Metabolism and Environmental Information Processing. Metabolism accounted for a dominant 78.3%, indicating that sodium butyrate exerts a broad regulatory effect on the body’s basal metabolic activities. Specifically, the metabolic pathways regulated by sodium butyrate included lipid metabolism, global and overview profiles, amino acid metabolism, and cofactor and vitamin metabolism. The Environmental Information Processing category involved a small number of differentially expressed metabolites, primarily related to signal transduction. Notably, the “Tuberculosis” pathway showed significant enrichment in the positive ion mode. However, because this KEGG category includes numerous immune- and inflammation-related signaling components that are shared across multiple biological processes, its enrichment should not be interpreted as evidence of tuberculosis-related alterations. Rather, it may reflect changes in immune-metabolic regulation associated with sodium butyrate supplementation. Therefore, greater emphasis was placed on pathways more directly related to lipid metabolism, amino acid metabolism, and inflammatory regulation. In addition, metabolic pathways such as steroid biosynthesis, the sphingolipid signaling pathway, and arachidonic acid metabolism also exhibited significant differences. It should be noted that the biological interpretation of these pathways in avian species remains relatively limited compared with mammalian systems. Therefore, the enrichment of sphingolipid signaling and arachidonic acid metabolism pathways should be interpreted as evidence of altered lipid-related and immune-related metabolic processes rather than as direct indicators of specific physiological outcomes. In birds, metabolites involved in these pathways are known to participate in membrane structure maintenance, cellular signaling, oxidative stress responses, and inflammatory regulation. However, the precise functional significance of these pathway alterations in tumbler pigeons requires further investigation. Sphingolipids are important components of cell membranes and participate in various biological processes such as cell growth, differentiation, and apoptosis [107]. In addition, arachidonic acid metabolism and α-linolenic acid metabolism are closely associated with inflammatory regulation and cellular homeostasis. The enrichment of these pathways is broadly consistent with the observed alterations in inflammatory cytokines and antioxidant indicators; however, the present data do not establish direct causal relationships between pathway enrichment and physiological responses. Collectively, these pathway alterations indicate that sodium butyrate supplementation was associated with changes in lipid metabolism and immune-related metabolic pathways, highlighting its potential role in maintaining physiological homeostasis under exercise-induced stress.

In the negative ion mode, differentially expressed metabolites were primarily distributed across two major categories: Organismal Systems and Metabolism. Among these, 1–2 differentially expressed metabolites were involved in Organismal Systems, specifically the sensory and digestive systems, suggesting that sodium butyrate supplementation may be associated with metabolic alterations in pathways classified within sensory and digestive system-related categories. Previous studies have shown that microbial metabolites can influence multiple host physiological processes, including immune and metabolic regulation [108]. In the Metabolism category, differentially expressed metabolites were more widespread, encompassing multiple key pathways including global and overview profiles, amino acid metabolism, nucleotide metabolism, lipid metabolism, carbohydrate metabolism, cofactor and vitamin metabolism, and other amino acid metabolism. In negative ion mode, the metabolic pathways showing the most significant differences between the sodium butyrate-treated group and the control group were 2-oxocarboxylic acid metabolism and biosynthesis of amino acids. The enrichment of 2-oxocarboxylic acid metabolism and amino acid biosynthesis pathways suggests that sodium butyrate supplementation was associated with alterations in central carbon metabolism and amino acid-related metabolic processes. These metabolic alterations may contribute to the maintenance of metabolic homeostasis during exercise stress and are consistent with the observed changes in antioxidant and inflammatory parameters. Previous studies have reported that butyrate serves as an important energy source for intestinal epithelial cells and may support intestinal function. The metabolic alterations observed in the present study are broadly consistent with the known physiological roles of butyrate. Furthermore, pyrimidine metabolism also exhibited high significance, suggesting that nucleotide-related metabolic processes may have been affected by sodium butyrate supplementation. Although the metabolic pathway (map01100) contained the largest number of differentially expressed metabolites, its statistical significance was relatively low, indicating that the overall perturbation of this pathway was mild. This observation is consistent with the role of sodium butyrate as a nutritional regulator rather than a potent pharmacological intervention. Collectively, the metabolomic results suggest that sodium butyrate may influence interconnected metabolic pathways involved in inflammatory regulation, antioxidant defense, and metabolic adaptation under exercise-related stress conditions.

Supplementation with sodium butyrate exerts a significant regulatory effect on the physiological homeostasis of tumbler pigeons under high-intensity flight stress, primarily manifested through its comprehensive influence on plasma metabolomics, blood gas homeostasis, antioxidant capacity, immune regulation, and inflammatory responses. Spearman’s r analysis revealed significant correlations between relevant blood gas parameters, antioxidant and immune markers, inflammatory factors, and plasma non-targeted differential metabolites. Regarding blood gas homeostasis, the metabolite hydroxyectoine showed a strong positive correlation with Cl^−^. As a compatible solute with dual osmoprotective and antioxidant functions, the upregulation of hydroxyectoine may enhance cellular osmotic regulation, stabilize transmembrane ion gradients, and thereby help maintain acid-base balance and oxygen transport efficiency. However, the causal relationship between hydroxyectoine metabolism and blood gas regulation was not directly investigated in the present study and warrants further research. In addition, the metabolite Mytilin A showed a strong negative correlation with PvO_2_ and a strong negative correlation with SvO_2_. Mytilin A is a natural antimicrobial peptide that plays a key role in the immune defense system; its expression levels are induced by pathogen stimulation [109]. The observed decrease in Mytilin A, together with its negative correlation with PvO_2_ and SvO_2_, suggests a potential association between this metabolite and exercise-related physiological responses in tumbler pigeons. These findings may indicate that sodium butyrate supplementation influences metabolic pathways associated with oxygen utilization and physiological adaptation during training and competition. However, the underlying mechanisms remain unclear and require further investigation.

Analysis of the correlation between plasma differential metabolites and antioxidant status revealed that GSH-PX showed a strong negative correlation with norepinephrine sulfate and lysine–tryptophan–lysine (Lys-Trp-Lys), while exhibiting a strong positive correlation with (3R)-3-hydroxy-12′-apo-beta-carotene. Norepinephrine sulfate, a salt form of norepinephrine, activates multiple cellular receptors and has the effects of raising blood pressure, enhancing myocardial contractility, and increasing heart rate [110]. The negative correlation observed between GSH-PX and norepinephrine sulfate suggests a potential relationship between antioxidant responses and catecholamine-related metabolic activity following sodium butyrate supplementation. Lys-Trp-Lys, a cationic, tryptophan-rich tripeptide, possesses certain antibacterial and antioxidant capabilities [111]. Under conditions of oxidative stress, a similar negative correlation exists between antioxidant metabolites and enzymatic antioxidant markers; in such cases, increased antioxidant enzyme activity is typically accompanied by changes in the utilization and metabolic turnover of low-molecular-weight antioxidant compounds. Therefore, the decrease in Lys–Trp–Lys concentration may reflect a shift in antioxidant-related metabolic processes as the body adapts to exercise-induced oxidative stress, rather than indicating that the peptide itself has a direct regulatory effect. (3R)-3-hydroxy-12′-apo-beta-carotene is a deprotonated carotenoid and an important antioxidant capable of scavenging singlet oxygen and free radicals, thereby alleviating oxidative stress [112]. The positive correlation between this metabolite and GSH-PX suggests that enhanced enzymatic antioxidant defense capacity may occur concurrently with increased carotenoid-related antioxidant metabolism. Ruixia, L. et al. [113] found in a study on broiler chickens that the addition of sodium butyrate may alleviate heat stress-induced structural and functional dysfunction of the jejunal tight junctions by upregulating the expression of claudin, tight junction protein-1, and ZO-1, thereby reducing oxidative stress and inflammatory responses. Although intestinal barrier function and antioxidant signaling pathways were not directly evaluated in the present study, the combined changes in antioxidant indicators and metabolite profiles suggest that sodium butyrate supplementation may contribute to improved redox homeostasis in tumbler pigeons. However, whether these effects involve modulation of mitochondrial function, carotenoid metabolism, Nrf2-related signaling pathways, or other antioxidant regulatory mechanisms remains to be clarified through targeted mechanistic studies.

With regard to the immune system, IgG shows a strong negative correlation with phenylglucuronide and 3-(3,4,5-trimethoxyphenyl)propanoic acid. Phenylglucuronides are an important class of Phase II metabolites formed through the glucuronidation process. They play a key role in the detoxification and clearance of exogenous and endogenous compounds in the body and constitute a major detoxification pathway for the elimination of phenol and its derivatives [114]. Elevated phenylglucuronide levels have been reported to be associated with increased detoxification demands or the accumulation of metabolites derived from intestinal microbial activity. In the present study, the negative correlation between IgG and phenylglucuronide suggests a potential association between this metabolite and immune-related responses following sodium butyrate supplementation. However, the underlying mechanisms remain unclear, and the present study did not directly evaluate detoxification pathways, UGT enzyme activity, or gut–liver axis function. 3-(3,4,5-Trimethoxyphenyl)propanoic acid is an aromatic propanoic acid derivative; its derivatives and structurally similar compounds possess certain biological activities, such as antitumor [115] and anti-inflammatory [116] effects. The strong negative correlation observed between this metabolite and IgG may indicate a potential relationship between aromatic metabolite metabolism and immune responses. Nevertheless, whether this metabolite directly participates in immune regulation requires further investigation.

Analysis of the correlation between differential metabolites and inflammatory factors in plasma revealed that IL-6 showed a very strong positive correlation with chlorothalonil-4-hydroxy, while exhibiting a very strong negative correlation with 3β-hydroxy-5-cholenoic acid. Furthermore, the anti-inflammatory factor IL-10 showed a strong positive correlation with orotidine and a strong positive correlation with phenylglucuronide. Chlorothalonil-4-hydroxy is a metabolite associated with environmental exposure [117]. In the present study, its positive correlation with IL-6 suggests a potential association between environmental-derived metabolites and inflammatory responses; however, the source of this metabolite and its biological significance in pigeons remain unclear. In the sodium butyrate intervention group, IL-6 levels in the pigeons decreased significantly as the dose of sodium butyrate increased, indicating that the anti-inflammatory effect of sodium butyrate can effectively alleviate the subclinical inflammatory state in pigeons caused by the combined effects of environmental factors and exercise stress. In addition, orotidine is a key intermediate in the de novo synthesis pathway of pyrimidine nucleotides, which are components of DNA and RNA and are essential for cell proliferation, growth, and various metabolic processes [118]. The observed positive correlation between IL-10 and orotidine suggests a potential association between nucleotide-related metabolic activity and anti-inflammatory responses in tumbler pigeons. These findings suggest that sodium butyrate supplementation is associated with metabolic changes linked to immune regulation; however, the underlying mechanisms were not directly examined in the present study and warrant further investigation.

Several limitations of the present study should be noted. First, the metabolomic analysis was performed exclusively on samples from the CON and T2 groups. While T2 was selected for its representative physiological responses, the identified metabolites and enriched pathways should be interpreted as potential metabolic shifts rather than definitive biomarkers for the optimal dose. Second, the exact sex of the pigeons was neither determined nor balanced during experimental allocation, meaning potential sex-related differences cannot be excluded; therefore, these findings reflect the overall response of a mixed-sex population. To address these limitations, future studies should employ larger sample sizes, evaluate sex as an independent variable, and incorporate multiple supplementation levels with orthogonal polynomial regression to fully characterize the linear and nonlinear dose-dependent metabolic responses.

## 5. Conclusions

In summary, dietary supplementation with sodium butyrate improved nutrient digestibility, enhanced antioxidant and anti-inflammatory responses, helped maintain blood gas balance and acid–base homeostasis, and was associated with changes in plasma metabolic profiles in tumbler pigeons. Among the tested supplementation levels, sodium butyrate elicited dose-dependent physiological responses, with the higher supplementation level (18 mg/d) generally showing stronger effects on antioxidant, immune, and inflammatory parameters.

The present findings indicate that sodium butyrate supplementation was associated with changes in nutrient utilization, physiological parameters, and metabolic responses in tumbler pigeons subjected to regular training and competition. These results expand current knowledge regarding the physiological and metabolic adaptations associated with sodium butyrate supplementation and provide a scientific basis for future nutritional management strategies in competitive pigeons. Further studies are required to determine whether these physiological changes translate into measurable improvements in athletic performance. In addition, the metabolomic analysis identified several pathways potentially associated with sodium butyrate supplementation; however, the underlying biological mechanisms remain to be confirmed through targeted mechanistic investigations.

## Figures and Tables

**Table 1:** Basic Diet Composition and Nutritional Levels for tumbler Pigeons (Dry Matter Basis).

Composition	Content %	Nutritional Index	Nutritional Status
Hulled barley	34.00	DM (%)	89.53
Hulled rice	24.90	OM (%)	97.64
Safflower seeds	8.65	GE (MJ)	16.98
Red sorghum	7.75	CP (%)	18.34
Brown rice	7.05	EE (%)	15.31
Wheat	6.60	NDF (%)	24.90
White sorghum	4.00	ADF (%)	2.64
Mung beans	2.05	Ca (%)	0.38
Hulled oats	2.05	P (%)	0.21
White glutinous rice	1.45		
Five-color millet	0.95		
Rapeseed	0.30		
Flaxseed	0.25		
Total	100		

**Table 2:** Effects of sodium butyrate supplementation on the apparent nutrient metabolism rates of tumbler pigeons.

Items	CON	T1	T2	T3	*p*
DM %	70.34 ± 1.90 ^b^	71.67 ± 2.25 ^ab^	73.76 ± 0.71 ^a^	73.05 ± 0.61 ^a^	0.014
OM %	78.86 ± 1.88	79.29 ± 1.88	80.54 ± 0.47	80.09 ± 1.58	0.352
CP %	44.50 ± 6.55	44.60 ± 4.37	43.08 ± 2.06	46.73 ± 6.30	0.737
EE %	84.24 ± 3.25	86.55 ± 1.96	84.52 ± 6.54	88.94 ± 2.51	0.254
ME MJ/kg	13.42 ± 0.30 ^Bc^	13.44 ± 0.36 ^Bc^	13.98 ± 0.34 ^ABb^	14.45 ± 0.22 ^Aa^	<0.001

Note: Values within the same row with no superscript letters or identical superscripts indicate no significant difference (*p* > 0.05). Values with different lowercase letters indicate significant differences (^a^, ^b^, ^c^: *p* < 0.05), and those with different uppercase letters indicate highly significant differences (^A^, ^B^: *p* < 0.01). Data are presented as mean ± standard deviation (*n* = 10).

**Table 3:** Plasma biochemical, antioxidant, immune, inflammatory, lipid metabolism, and enzyme activity parameters in tumbler pigeons supplemented with sodium butyrate.

Items	CON	T1	T2	T3	*p*
TP (g/L)	29.23 ± 3.63	25.92 ± 2.28	29.13 ± 2.07	27.45 ± 3.44	0.051
ALB (g/L)	15.71 ± 1.74 ^a^	14.00 ± 1.78 ^b^	15.69 ± 0.94 ^a^	15.59 ± 0.93 ^a^	0.026
GLB (g/L)	13.52 ± 3.80	11.92 ± 3.15	13.45 ± 2.27	11.86 ± 2.74	0.444
A/G	1.23 ± 0.29	1.26 ± 0.38	1.20 ± 0.23	1.35 ± 0.19	0.652
UA (umol/L)	371.43 ± 139.24	319.84 ± 70.64	413.47 ± 137.50	411.45 ± 108.15	0.257
SOD (U/mL)	60.78 ± 7.63 ^Cc^	67.24 ± 6.70 ^BCc^	77.16 ± 10.62 ^Bb^	88.40 ± 10.92 ^Aa^	<0.001
CAT (U/mL)	31.70 ± 7.21 ^Cc^	41.14 ± 2.93 ^Bb^	44.63 ± 50 ^Bb^	51.51 ± 6.46 ^Aa^	<0.001
GSH-PX (U/mL)	120.52 ± 12.63 ^Cd^	143.95 ± 16.33 ^Bc^	157.70 ± 8.99 ^ABb^	170.25 ± 12.09 ^Aa^	<0.001
MDA (nmol/mL)	3.53 ± 1.07	3.19 ± 0.66	2.92 ± 1.02	2.79 ± 0.53	0.236
T-AOC (U/mL)	6.66 ± 0.54 ^Dd^	7.49 ± 0.29 ^Cc^	8.46 ± 0.31 ^Bb^	9.42 ± 0.31 ^Aa^	<0.001
IgM (g/L)	1.33 ± 0.25	1.59 ± 0.44	1.60 ± 0.23	1.69 ± 0.4	0.116
IgG (g/L)	4.77 ± 1.06	4.06 ± 1.15	4.55 ± 0.67	4.82 ± 0.68	0.255
IgA (g/L)	2.55 ± 0.9	2.10 ± 0.34	2.49 ± 0.84	2.85 ± 1.77	0.507
IL-4 (pg/mL)	3.51 ± 0.7 ^Cd^	4.38 ± 1.10 ^Cc^	5.53 ± 0.61 ^Bb^	8.00 ± 0.72 ^Aa^	<0.001
IL-10 (pg/mL)	8.27 ± 1.01 ^Cd^	10.43 ± 1.69 ^Bc^	12.44 ± 2.02 ^Bb^	15.89 ± 2.15 ^Aa^	<0.001
γIFN (pg/mL)	41.29 ± 4.97 ^Aa^	36.18 ± 5.85 ^AaBb^	32.53 ± 8.05 ^BbC^	26.11 ± 3.28 ^Cc^	<0.001
IL-1β (pg/mL)	28.88 ± 6.72 ^Aa^	25.27 ± 6.65 ^AaBb^	21.71 ± 2.47 ^BbC^	16.05 ± 3.01 ^Cc^	<0.001
IL-6 (pg/mL)	152.05 ± 9.79 ^Aa^	135.85 ± 7.50 ^Bb^	125.83 ± 4.68 ^Bc^	113.64 ± 14.03 ^Cd^	<0.001
IL-8 (pg/mL)	87.18 ± 6.53 ^Aa^	73.13 ± 2.57 ^Bb^	63.22 ± 9.17 ^Cc^	51.36 ± 8.43 ^Dd^	<0.001
IL-18 (pg/mL)	91.13 ± 14.75 ^a^	85.69 ± 8.91 ^a^	78.92 ± 14.10 ^ab^	72.05 ± 16.10 ^b^	0.022
TNFα (pg/mL)	64.09 ± 27.33 ^a^	58.33 ± 4.62 ^b^	52.34 ± 6.70 ^ab^	43.85 ± 6.79 ^b^	0.026
TC (mmol/L)	8.93 ± 1.57 ^bc^	8.31 ± 1.44 ^c^	9.53 ± 0.51 ^ab^	10.16 ± 1.04 ^a^	0.011
TG (mmol/L)	2.18 ± 0.63 ^AaBb^	1.70 ± 0.44 ^Bb^	2.72 ± 0.66 ^AaB^	2.52 ± 0.88 ^Aa^	0.009
GLU (mmol/L)	18.37 ± 1.17 ^b^	18.31 ± 1.71 ^b^	19.76 ± 1.67 ^a^	19.87 ± 1.05 ^a^	0.024
LAC (mmol/L)	3.53 ± 1.07	3.20 ± 0.66	2.92 ± 1.02	3.49 ± 1.34	0.541
AST (U/L)	156.99 ± 44.22 ^ABb^	136.15 ± 26.02 ^Bb^	208.12 ± 72.88 ^Aa^	211.46 ± 54.22 ^Aa^	0.004
ALT (U/L)	30.48 ± 7.2	28.18 ± 8.13	35.40 ± 8.21	33.87 ± 9.57	0.221
ALP (U/L)	794.50 ± 400.37	746.32 ± 306.17	571.16 ± 139.2	770.21 ± 346.61	0.383
γ-GT (U/L)	3.02 ± 1.22	4.13 ± 0.92	3.61 ± 1.31	3.54 ± 0.96	0.191
LDH (U/L)	116.70 ± 66.18 ^Aa^	55.15 ± 14 ^Bb^	74.51 ± 40.26 ^ABb^	65.82 ± 18.27 ^ABb^	0.009
CK (U/L)	794.63 ± 359.35	679.97 ± 405.9	844.03 ± 519.38	918.37 ± 568.1	0.717

Note: Values with different lowercase letters indicate significant differences (^a^, ^b^, ^c^, ^d^: *p* < 0.05), and those with different uppercase letters indicate highly significant differences (^A^, ^B^, ^C^, ^D^: *p* < 0.01). Data are presented as mean ± standard deviation (*n* = 10).

## Data Availability

The data that support the findings of this study are available from the corresponding author upon reasonable request.

## Supplementary Materials

The following supporting information can be downloaded at https://www.mdpi.com/article/10.3390/ani16131941/s1, Table S1: Pre- and post-race performance data sheets for tumbler pigeons. Table S2: Effects of sodium butyrate on blood gas profiles in tumbler pigeons pre- and post-race. Table S3: Five significantly up- and downregulated metabolites in pigeon plasma (positive and negative ion modes).

## Abbreviations

The following abbreviations are used in this manuscript:

PvCO_2_	Partial pressure of carbon dioxide
PvO_2_	Venous oxygen partial pressure
TCO_2_	Total carbon dioxide
SvO_2_	Venous oxygen saturation
HCO_3_^−^	Bicarbonate ion
BEecf	Base excess of extracellular fluid
BEb	Base excess
Na^+^	Sodium ion
K^+^	Potassium ion
Ca^2+^	Calcium ion
Cl^−^	Chloride ion
Hb	Hemoglobin
Hct	Hematocrit
DM	Dry matter
OM	Organic matter
ME	Metabolizable energy
GE	Gross energy
CP	Crude protein
EE	Ether extract
CF	Crude fiber
NDF	Neutral detergent fiber
ADF	Acid detergent fiber
TP	Total protein
ALB	Albumin
GLB	Globulin
AST	Aspartate transaminase
ALT	Alanine transaminase
ALP	Alkaline phosphatase
UA	Uric acid
TC	Total cholesterol
TG	Triglycerides
GLU	Glucose
LAC	Lactate
LDH	Lactate dehydrogenase
γ-GT	γ-Glutamyltransferase
CK	Creatine kinase
MDA	Malondialdehyde
SOD	Superoxide dismutase
GSH-Px	Glutathione peroxidase
T-AOC	Total antioxidant capacity
CAT	Catalase
IgG	Immunoglobulin G
IgA	Immunoglobulin A
IL-4	Interleukin-4
IL-6	Interleukin-6
IL-8	Interleukin-8
IL-10	Interleukin-10
IL-18	Interleukin-18
IL-1β	Interleukin-1β
γ-IFN	Interferon-γ
TNF-α	Tumor necrosis factor-α
LC-MS	Liquid chromatography–mass spectrometry
PCA	Principal Component Analysis
PLS-DA	Partial Least Squares Discriminant Analysis
HCA	Hierarchical Cluster Analysis
HRMS	High-resolution mass spectrometry
CV	Coefficient of variance
ANOVA	Analysis of variance
SGLT1	Sodium–glucose cotransporter 1
PepT1	Peptide transporter 1
Lys-Trp-Lys	Lysine–tryptophan–lysine
